# Diet, Physical Activity and Gestational Weight Gain Patterns among Pregnant Women Living with Obesity in the North East of England: The GLOWING Pilot Trial

**DOI:** 10.3390/nu13061981

**Published:** 2021-06-09

**Authors:** Nicola Heslehurst, Angela C. Flynn, Lem Ngongalah, Catherine McParlin, Kathryn V. Dalrymple, Kate E. Best, Judith Rankin, Elaine McColl

**Affiliations:** 1 Faculty of Medical Sciences, Population Health Sciences Institute, Newcastle University, Newcastle upon Tyne NE1 7RU, UK; lem.ngongalah@newcastle.ac.uk (L.N.); judith.rankin@newcastle.ac.uk (J.R.); elaine.mccoll@newcastle.ac.uk (E.M.); 2 Department of Women and Children’s Health, Kings College London, Strand, London WC2R 2LS, UK; angela.flynn@kcl.ac.uk (A.C.F.); kathryn.dalrymple@kcl.ac.uk (K.V.D.); 3 Faculty of Health and Life Sciences, Northumbria University, Sutherland Building, Newcastle upon Tyne NE1 8ST, UK; catherine.mcparlin@northumbria.ac.uk; 4 Leeds Institute of Health Sciences, University of Leeds, Woodhouse, Leeds LS2 9JT, UK; k.e.best@leeds.ac.uk

**Keywords:** pregnancy, obesity, diet, physical activity, gestational weight gain, deprivation

## Abstract

Maternal diet, physical activity (PA) behaviours, and gestational weight gain (GWG) are important for optimum health of women and their babies. This secondary analysis of the GLOWING pilot cluster trial explored these among women living with obesity in high deprivation. Pregnant women completed food frequency, PA and psychosocial questionnaires. Weights were retrieved from medical records and measured during routine appointments with midwives. Descriptive and regression analyses were stratified by obesity class. A total of 163 women were recruited; 54.0% had class 1 obesity, 25.8% class 2, 20.2% class 3, and 76.1% lived in the two most deprived quintiles. Women had suboptimal dietary intake, particularly for oily fish, fruit and vegetables. PA was predominantly light intensity, from household, care and occupational activities. Most women gained weight outside of Institute of Medicine (IOM) guideline recommendations (87.8%); women in class 3 obesity were most likely to have inadequate GWG below IOM recommendations (58.3%, *p* < 0.01) and reduced odds of excessive GWG compared with class 1 (AOR 0.13, 95% 0.04–0.45). Deprived women with obesity have a double inequality as both increase pregnancy risks. This population requires support to meet guideline recommendations for diet, PA and GWG. Further research exploring obesity classes would inform policies and care to achieve the best pregnancy outcomes.

## 1. Introduction

In the UK, approximately one in five women who access maternity services have a body mass index (BMI) in the obese range (BMI ≥ 30 kg/m^2^) [1], with the odds of obesity being up to five-fold higher among women living in areas of highest versus least deprivation [2]. The short- and long-term risks of maternal obesity are multiple and severe, including gestational diabetes (GDM), pre-eclampsia, maternal and offspring mortality [3,4,5], and a 264% increase in the odds of childhood obesity in offspring [6], which may be in part due to nutritional epigenetic changes in utero [7]. However, pregnancy is an opportunity for intervention to improve maternal diet and physical activity (PA) behaviours and limit gestational weight gain (GWG) which can reduce the risk of GDM, hypertensive disorders of pregnancy, and caesarean section, and improve maternal cardiorespiratory fitness [8,9,10,11]. Providing weight management support can improve women’s diet and PA behaviours during pregnancy and postnatally, and significantly reduce GWG and postnatal weight retention [12,13,14]. 

The UK National Institute of Health and Care Excellence guidance for maternal diet includes eating five portions of fruit and vegetables a day, and one portion of oily fish a week. It also states that energy needs do not change in the first 6 months of pregnancy and increase only by 200 calories per day in the last 3 months, and recommends daily supplements including folic acid and vitamin D [15,16]. UK recommendations for PA in pregnancy include aiming to achieve at least 150 min of moderate-intensity PA each week, muscle-strengthening activities twice a week and breaking up prolonged periods of sedentary time [17]. There are currently no national GWG guidelines in the UK other than recommendations that women should not try to reduce obesity-related risks by “dieting” (i.e., losing weight) during pregnancy, and they should be encouraged to lose weight after pregnancy [18]. The USA Institute of Medicine (IoM) GWG guidelines have been widely adopted internationally and recommend that women with an obese preconception BMI should aim for a total GWG between 5 and 9 kg [19]. Total GWG includes 0.5–2 kg in the first trimester, and a mean weekly GWG of 0.22 kg (range 0.17–0.27 kg) in the second and third trimesters [19]. Research into maternal obesity often groups all women with a BMI over 30 kg/m^2^ together. However, there is a growing body of evidence showing that obesity-associated risks are greater in higher obesity classes [20,21,22], suggesting that the IoM guidelines should also be stratified by obesity class to reflect differences in risk. A meta-analysis of almost 740,000 women living with obesity has suggested that current recommendations may only be applicable to women with class 1 obesity (BMI 30.0–34.9 kg/m^2^), and that a lower total GWG of 1 to <5 kg for women with class 2 obesity (BMI 35.0–39.9 kg/m^2^) and 0 kg GWG for women with class 3 obesity (≥40 kg/m^2^) could improve foetal growth and caesarean delivery outcomes [23]. More recently, an observational study using routine data for 337,590 women in Belgium reported that a total GWG of between 0 kg (class 1 obesity) and −5 kg (class 3 obesity) was associated with a reduced risk of a range of outcomes including hypertension, emergency caesarean and high- and low-birth-weight outcomes [24]. An individual patient data meta-analysis of 196,670 women from 25 cohort studies across Europe and North America reported a reduced risk of one or more adverse pregnancy outcomes with GWG between 2 kg and <6 kg for class 1 obesity, a weight loss or GWG of up to 4 kg for class 2 obesity, and GWG between 0 kg and <6 kg for class 3 obesity [25]. However, caution is needed in interpreting these results due to a lack of prospective evidence to demonstrate the safety of weight loss in pregnancy.

Despite the significantly increased risks associated with obesity and the strong association with deprivation, there are limited data exploring patterns of diet, PA and GWG among women living with obesity in deprived populations. There is also an absence of data which explores whether there are any differences in patterns between obesity classes. These data would enable us to compare patterns with guideline recommendations, identify target areas for improvement and future interventions for women with the highest level of risk and facing the highest levels of inequality. The aim of this study was to explore the patterns of diet and PA behaviours, and GWG among pregnant women living with obesity in a highly deprived region of England. Additionally, we explored whether there were any differences in these patterns between obesity classes. 

## 2. Materials and Methods

This study is a secondary analysis of data collected during the GestationaL Obesity Weight management: Implementation of National Guidelines (GLOWING) pilot trial. The published protocol reports the description of the intervention and pilot trial methods [26]. GLOWING was developed using social cognitive theory in order to support midwives’ implementation of national guidelines for weight management during pregnancy, and delivered as an intensive midwife training day plus provision of resources for routine practice [26]. Primary intervention outcomes related to change in midwifery practice. The intervention was piloted as a cluster RCT in four National Health Service (NHS) Trusts in the North East of England, UK, a region which includes some of the most deprived localities in England [27] and has a significantly higher than national average prevalence of maternal obesity [2]. Pregnant women were recruited to GLOWING for data collection purposes only and did not directly receive any intervention. Recruitment took place during routine ultrasound scan appointments in the four participating NHS Trusts. Women were eligible if they had a booking BMI ≥ 30.0 kg/m^2^, were aged 18 or over, and could speak and read English (due to the lack of translated validated questionnaires). All recruited women were asked to complete questionnaires with components on their socio-demographics; the types of discussions they had with their midwives about weight, diet and PA; their diet and PA behaviours; and psychosocial questions relating to their weight. Different time points and recruitment strategies were used pre- and post-intervention delivery to test trial data collection procedures for a future definitive trial. Prior to delivering the GLOWING intervention to midwives, the recruitment strategy involved randomising women who had their first antenatal appointment at a GLOWING NHS Trust and approaching women at their 20 week scan appointment to complete a one-off questionnaire including all components; we aimed to recruit 15 women per cluster (60 in total) at this stage (sample 1). After delivering the intervention to midwives, the recruitment strategy involved convenience sampling, where any women who were booked for a 12 week scan who met the inclusion criteria were approached for recruitment (sample 2). For sample 2, we aimed to recruit 60 women at 12 weeks gestation to provide questionnaire data on the types of discussions they had with their midwife during their first antenatal contact, and to follow up these women in their third trimester, at approximately 36 weeks gestation, to complete a second questionnaire on their diet and PA behaviours. Sample size was determined based on recommendations for pilot trials [26]. Women received a £10 gift voucher for each questionnaire they returned. The pilot trial was not powered to detect any between group differences, and analysis exploring any potential intervention effect confirmed this (Table S1). Therefore, the STROBE guidelines for reporting observational studies [28] have been used (Table S2). 

### 2.1. Socio-Demographic Data Collection

The socio-demographic questions included quantitative and free-text items relating to the women’s booking BMI, ethnic group, education, employment, marital status, smoking, alcohol intake, and other factors which may influence dietary patterns (e.g., whether a vegetarian diet was followed, whether women were experiencing nausea or vomiting, and food aversions or cravings). Booking weight, height and BMI were also retrieved from the women’s routine medical records and the WHO criteria [29] for obesity classes 1–3 were applied. Women’s postcodes were linked to the Index of Multiple Deprivation (IMD) ranks of deprivation (1 to 32,844) [30]. The IMD ranks were grouped into quintiles of equal proportion to determine deprivation status, where quintile 1 was the most deprived and quintile 5 was least deprived. 

### 2.2. Dietary Data Collection

Dietary data collection used a semi-quantitative food frequency questionnaire (FFQ) that was developed for the UK UPBEAT study for women with obesity [31]. The FFQ included 50 items and was originally adapted from the UK arm of the European Prospective Investigation into Cancer (EPIC) Study [32]. The FFQ items included a combination of free-text responses (e.g., brand and average consumption per month of bread, breakfast cereal, butter/spread and cheese), as well as a multiple response grid for participants to report frequency of food item consumption over the preceding month (ranging from 1: never/less than once per month to 9: six or more per day) [31]. The FFQ was validated against 24 h recalls collected during the UPBEAT pilot study among women with an obese BMI [33]. To convert frequency of consumption to daily nutrient intakes, average portion sizes were utilised and conversion factors applied [31]. The 50 FFQ items were grouped into dietary categories and subcategories for analysis (Table S3). 

### 2.3. Physical Activity Data Collection

PA data collection used the Pregnancy PA Questionnaire (PPAQ), a validated self-report tool which covers 32 questions grouped into different modes of PA: household/caregiving (13 questions), occupational (five questions), sports/exercise (eight questions), transportation (three questions) and inactivity (three questions) [34]. Women selected one of six options for time spent doing each activity per day or per week. Each type of activity has a specific metabolic equivalent task (MET) value allocated to it using field-based measurements in pregnant women [35] and the PA compendium-based MET values (1 MET = 1 kcal/kg × hour) [36]. The MET values were used to classify each activity by intensity—sedentary (≤1.5 METs), light (1.5–3.0 METs), moderate (3.0–6.0 METs) or vigorous (>6.0 METs)—and the average number of MET-hours per week expended in each intensity level was calculated. Overall estimated energy expenditure (EE) was calculated by multiplying duration by intensity of activity. PPAQ coding instructions were obtained from the original research team and are available online [37]. Outcomes reported are for total EE, PA mode and intensity (Table S4).

### 2.4. Gestational Weight Gain Data Collection

A clinical audit was carried out to explore routine recording of weight measurements throughout pregnancy for all women participating in GLOWING. Women in sample 2 were also given a weight card for their midwife to complete at their routine 36 week appointment. We used the IoM [19] guidelines as reference criteria to assess adequacy of GWG, where GWG within the recommendations was considered to be adequate, below the recommendations was inadequate, and above was excessive. However, using the total GWG recommendations of 5–9 kg as reference criteria requires both a preconception weight and a weight at delivery; these measures are not usually routinely collected [38]. Instead, researchers and clinicians rely on weights measured in the first, second and third trimesters of pregnancy. Applying the IoM total GWG criteria to estimate adequacy of GWG when using different time points for weight measurements can result in inaccurate interpretations. For example, in the UK the first pregnancy weight is measured at the booking appointment which is usually in the first trimester. Therefore, booking weights already include some, or all, of the 0.5–2 kg GWG factored into the IoM total GWG recommendations. Additionally, the total GWG time period from conception to delivery assumes a full-term pregnancy, and as women can deliver earlier (or later) than 40 weeks, the appropriate GWG ranges should be adjusted for gestational age [38]. Despite recognition of the challenges with estimating adequacy of GWG when using routine weight measurements taken at varying time points during pregnancy, there is currently no agreed approach for handling these challenges. We developed a novel approach to determining adequacy of GWG based on published comparisons of methods [38]. We reviewed the booking records for all women over a 1-year period in the four NHS Trusts participating in GLOWING. The mean gestational age at booking was 11.2 weeks (SD 4.1). Therefore, we assumed the booking weights for most women in this study already included the first trimester GWG factored into the IoM recommendations (0.5–2 kg). We calculated the recommended GWG for each week of pregnancy beyond the first trimester (from week 13) using the IoM obesity-specific mean and range for trimesters two and three (Table S5). The weights measured at the latest gestational age in the second or third trimester were used to determine whether that gestation-specific GWG was adequate, inadequate or excessive for women in GLOWING, according to IOM guidelines. Due to a very small proportion of women in the adequate GWG category, the three categories were collapsed to explore patterns in excessive versus non-excessive GWG. We compared our approach of determining excessive GWG at any gestational age in the second or third trimester, with an alternative approach of only including women with a weight measurement at 36 weeks to determine excessive GWG, to explore any differences between these two approaches in the analysis. 

### 2.5. Psychosocial Measures Relating to Weight-Related Behaviours

Women completed a validated questionnaire exploring psychosocial measures for understanding weight-related behaviours in pregnancy [39]. The questionnaire is validated for use in any trimester of pregnancy [39], and the women participating in GLOWING completed it at 20 weeks gestation (sample 1) and at 12 weeks gestation (sample 2). This questionnaire was developed using psychological theories (including social cognitive theory and theory about how changes in social roles influence psychological characteristics and health behaviours) and an in-depth interview study [39]. The constructs include 49 questions in total, relating to weight locus of control (four questions); body image (four questions); self-efficacy related to food intake, controlling weight, and performing regular exercise (eight questions); attitudes about weight gain during pregnancy (13 questions); feelings about motherhood (seven questions); and career orientation (13 questions). All items were assessed using three-, four- or five-point Likert scales. The scales were dichotomized or trichotomized as appropriate into categories of agreement and disagreement (plus neither agree nor disagree for 5-point scales) for descriptive analysis [40]. Scales were summed, following reverse coding of items where required [40] to compare the socio-cognitive factors between obesity classes Data analysis was carried out for all GLOWING participants combined, with subgroup analysis exploring any differences in reporting behaviours due to the different gestational ages of questionnaire data collection (samples 1 and 2). Descriptive analysis was carried out for population characteristics, and patterns in diet, PA and GWG. Outcome data were tested for normality of distribution (Shapiro-Wilkes test). Means and standard deviations (SD) are presented for normally distributed data alongside p-values derived from t-tests and ANOVA. Median and interquartile ranges (IQR) are presented for non-normally distributed data alongside p-values derived from Mann-Whitney U tests and Kruskal-Wallis tests. Chi squared tests were used to explore patterns in categorical outcome data. Logistic regression was carried out to explore whether obesity class was associated with excessive GWG. The model estimated the odds of excessive GWG for obesity classes 2 and 3 compared with obesity class 1 and included binary variables for intervention arm of the pilot trial, and sample 1 or 2. 

There were different types of missing data in the dataset; (1) questionnaires that were completely missing and therefore no diet or PA data reported for those women, (2) questionnaires that had been returned but where there were some FFQ and PPAQ questions that had not been completed, (3) women with no follow up weight measurements, and (4) women with some follow up weight measurements but not for 36 weeks’ gestation. For all types of missing data, we considered whether multiple imputation was appropriate. For the completely missing diet and PA data (1), multiple imputation was not appropriate as the number of variables required in the imputation model (FFQ 50 items and PPAQ 32 items) was greater than 1/3 of the complete data available [41]. The number of cases with missing FFQ and PPAQ items within questionnaires returned (2) was low, and complete case analysis was the most appropriate method. For missing weight data at 36 weeks gestation (3 and 4), we compared the socio-demographic variables for the complete and missing datasets (reported in results) and it was assumed that data were missing at random (where missingness can be explained by differences in observed data [42]) and the number of variables required in the imputation model met the minimum requirements. However, it is also possible that the weight data were missing completely at random, in which case a complete case analysis would be most appropriate. Multiple imputation of maternal weight at 36 weeks was performed using chained equations with 20 iterations [43]. Maternal weight at 36 weeks was included as a predictor in the chained equations along with maternal booking weight, BMI, parity, deprivation quintile, age, NHS Trust, intervention arm, sample 1 or 2, weight at 28 weeks gestation, maternal education, employment and marital status. Regression models were performed for excessive GWG using both original (complete case analysis) and imputed data to compare results and explore our assumptions. All statistical analysis was carried out in SPSS (v26 and v27). 

## 3. Results

### 3.1. Participant Characteristics

There were 163 women in total recruited to GLOWING: 59 women in sample 1 and 104 in sample 2. The mean gestational age at the time of completing the questionnaire was 20.8 weeks (SD 2.1, Table 1) for sample 1 and 12.8 weeks (SD 1.3) for sample 2. Women had a mean BMI of 36.2 kg/m^2^ (SD 6.0), 54.0% had class 1 obesity, 25.8% class 2, and 20.2% class 3 (Table 1). Women were predominantly White (93.9%) which was a higher than the national average for pregnancy (76.8% [1]). The mean age was 29.2 years (SD 5.3) which is reflective of the national average [1]. The median number of previous pregnancies was 2, with a median of 1 previous pregnancy progressing beyond 24 weeks. Most women were living in areas of highest deprivation, with 76.1% residing in the two most deprived quintiles (54.0% in the most deprived and quintile and 22.1% in the second most deprived quintile), substantially higher than the national average (50% of pregnant women living in two most deprived quintiles; 27.3% and 22.7% respectively) [1]. There were 62.7% of women in some form of paid employment compared with a national average of 71.8% women in the UK [44]. However, only 39.3% of women were in full time employment, whereas 20.2% were in part-time employment and 25.8% were unemployed. The majority of women reported no change from their usual employment during pregnancy (81.6%). Approximately half of the women identified as being single (50.3%), similar to the national average for pregnant women [45]. There were 46% of women whose highest level of education was GSCE or lower; this is a higher proportion than the national average for highest educational attainment among women in the UK (35% [46]). The comparison of missing questionnaire and weight data showed no significant difference between population socio-demographics (Table S6). There were some socio-demographic differences between obesity classes (Table S7). Compared with obesity classes 1 and 2, a higher proportion of women with class 3 obesity were living in the most deprived quintiles, were unemployed, had lower levels of education and were less likely to be married (Table S7).

### 3.2. Dietary Behaviours

Dietary data were reported by 98 women in total (Table 2). The majority of women ate both meat and fish (82.7%), and were not currently drinking alcohol (98.0%) or smoking cigarettes (87.7%). While overall the majority of women were not suffering with nausea (59.2%) or vomiting (71.4%), there were more women who did have these conditions at 20 weeks gestation (40.7% and 28.8%, respectively) than 36 weeks (33.3% and 20.5%, respectively). The inverse pattern was observed for heartburn (87.2% at 36 weeks gestation compared with 62.7% at 20 weeks). Among the women who described suffering with either nausea, vomiting or heartburn, most were eating or drinking the same as before (44.2%) or less than before (40.3%). The majority of women did not have any cravings (72.4%) and aversions (71.4%), and had not started eating or drinking anything different during pregnancy (71.4%); however, almost half of all women (48%) reported that they had stopped consuming some foods or drinks during pregnancy. In the free-text descriptions for these questions, women described cravings as primarily being sugar-sweetened products, fruit, fruit juice and vegetables; these were reflected in the types of foods and drinks women described as starting to eat during pregnancy. Aversions were described as predominantly being towards meat or fish. This was reflected somewhat in the descriptions of foods and drinks women had stopped consuming, but overall these were more closely related to the advice on what should be avoided during pregnancy (e.g., caffeine, alcohol, specific cheeses and methods of cooking eggs or meat) and sugar-sweetened products. Women who completed their questionnaires at 36 weeks gestation were additionally asked whether they had seen a dietitian or been diagnosed with GDM and the majority had not (82.1% and 87.2%, respectively). The majority of women who did see a dietitian were those with a GDM diagnosis.

#### Dietary Intake Based on FFQ Data

The FFQ-reported dietary intake was available for 97 women (Table 3). Compared to national recommendations for maternal diet, intake of oily fish was low (median 0 g/day, IQR 0, 9) as was consumption of fruit and vegetables (median 207 g/day, IQR 106, 344; n = 2.6 servings), with women reporting consuming less than one serving of vegetables per day (median 75 g/day, IQR 38, 125). The majority of women reported consuming reduced fat milk (71.3%), while 63.8% consumed full-fat cheese. Over half of women reported consuming white bread (52.9%), while less than half reported consuming non-refined breakfast cereals (45.6%), although approximately ¼ of women did not eat breakfast cereal. When dietary intake was stratified by obesity class, bread intake was significantly higher in women with class 3 obesity compared to classes 1 and 2. Although not statistically significant, intake of sugar-sweetened beverages, sweet snacks and red meat was higher among women with class 3 obesity compared to classes 1 and 2. When examining the dietary data by gestational age, women who completed the FFQ at 36 weeks gestation reported consuming less fruit juice, and more sweet snacks and starchy carbohydrate foods, in particular bread and breakfast cereals (all *p* < 0.05) (Table S8).

### 3.3. Physical Activity Behaviours

Out of the 98 women who returned questionnaires with PA data, there were 93 (94.9%) complete cases with a response to all PPAQ variables; 54 (91.5%) women in sample 1 and 39 (100%) women in sample 2. Overall, women reported a median EE of 166 MET-hr/week (IQR 128.1, 249.2) (Table 4). The majority of EE was from light-intensity PA (median MET-hr/week 111.4, IQR 79.0, 149.9), followed by moderate-intensity PA (median MET-hr/week 82.1, IQR 45.7, 134.8) and sedentary-intensity PA (median MET-hr/week 14.9, IQR 7.4, 28.0), with limited EE reported for vigorous-intensity PA (median MET-hr/week 0.0, IQR 0.0, 0.8) (Table 4). The type of activities that made up the EE were primarily household/care and occupational PA (median MET-hr/week 79.2, IQR 41.4, 135.5 and 71.6, IQR 13.0, 114.9, respectively), followed by inactive PA, transport and sport (Table 4). For most PA variables, there was no statistically significant difference between women who completed their questionnaire at 20 and 36 weeks gestation (Table S9). However, women at 36 weeks were more likely to report EE from moderate-intensity PA (*p* = 0.04) and occupation- and transportation-related activities (*p* = 0.02, *p* = 0.04, respectively) (Table S9). 

There was no statistically significant difference in MET-hr/week between the three obesity classes for EE, or any PA intensity or mode (Table 4). However, there was a pattern for women with class 3 obesity to report lower median MET-hr/week for total EE, light or moderate intensity, and occupational mode of PA, and higher sedentary intensity and inactive mode of PA than obesity classes 1 and 2 (Table 4), particularly for the data collected at 36 weeks gestation (Table S9). These data may be clinically significant, suggesting that PA decline over the course of pregnancy could be higher for women with class 3 obesity than for other obesity classes. 

### 3.4. Gestational Weight Gain

The mean booking weight for all women was 99.5 kg (SD 17.4), which significantly increased with increasing obesity class (Table 5). The majority (86.5%) of participants reported that they had previously tried to lose 10 lbs of weight, with differences observed among obesity classes (no women with class 3 obesity answered “no” to this question). Among women who had previously attempted weight loss, most felt that their weight loss attempts had been somewhat or very successful (47.5% and 23.4%, respectively), with no differences between obesity classes. There were 90 women (55.2%) with a total of 115 weight measurements recorded in the second or third trimester (Figure S1). There was a lack of consistency in the gestational age at which weight was measured, although there was clustering around 28 and 36 weeks gestation. The median gestational age of the latest weight measurement used to determine the adequacy of gestation-specific GWG was 36 + 0 weeks (IQR 30 + 6, 36 + 0 weeks). 

According to IOM guideline recommendations, most women either gained excessive weight for their gestational age (50.0%) or inadequate weight (37.8%), with few women gaining weight within the adequate range (12.2%) (Table 5). There was a significant difference in the patterns according to obesity class (*p* = 0.001); most women in classes 1 and 2 gained excessive weight (61.4% and 63.6%, respectively) compared with a minority of women in class 3 obesity (16.7%) who predominantly had inadequate GWG below the IoM guideline recommendations (58.3%) (Table 5). There was no difference in odds of excessive gestation-specific GWG (measured at any gestational age) for women with class 2 obesity compared with class 1 obesity in either the unadjusted (OR 1.10, 95% CI 0.38, 3.18) or adjusted (AOR 1.04, 95% CI 0.36, 3.06) models (Table S10). However, there was a significant reduction in odds of excessive GWG for women with class 3 obesity compared with class 1, in both models (OR 0.13, 95% CI 0.37, 0.43; AOR 0.13, 95% CI 0.04, 0.45). When comparing the different methods used to determine excessive GWG, there was little difference in proportions or odds ratios in the complete case analysis using weight measured at any gestational age or when restricting to the subgroup of women with weights measured at 36 weeks gestation (Table S10). However, following multiple imputation of weight at 36 weeks, the proportions of women gaining excessive weight at 36 weeks gestation and the effect size and significance of the association for obesity class 3 in the regression model changed, although the direction of effect remained (Table S10).

### 3.5. Psychosocial Measures Relating to Weight and Related Behaviours

Psychosocial data were available for all 163 women; 59 reported at 20 weeks gestation and 104 reported at 12 weeks gestation. Overall, there was a strong pattern in the data for negative body image for both body weight and shape, with most women perceiving their body weight as too heavy, their body shape as being too big, and being dissatisfied with both (Table S11). There was also a consistent pattern for women to have a strong internal locus of control relating to their ability to control their weight, and in their self-efficacy relating to weight loss after pregnancy, and diet and PA behaviours. 

Most women reported positive feelings towards motherhood, but attitudes to weight gain and career orientation were mixed. Women tended to agree with statements that they worried they may “get fat” and that they could not totally control GWG, but disagreed with statements that they were trying to keep their weight down so they did not look pregnant and that they liked being able to gain weight for a change. In relation to preferences towards career or family orientation, preferences were in the direction of career for eight out of 13 questions, and towards family orientation for the remaining five questions, suggesting that both career and family roles were important for women in this sample. There was limited difference for any items when comparing the gestational ages at time of completion of the questionnaire (Table S11).

There was a statistically significant difference between obesity classes for the body image scale, where women in the class 3 obesity group reported lower scores (indicating more negative body image) than classes 1 and 2 (Table 6). There was no statistically significant difference between obesity classes for the locus of control, self-efficacy, attitudes to weight gain, feelings about motherhood, or career orientation scales (Table 6). However, women with class 3 obesity had slightly higher scores for attitudes to weight gain (indicating more positive attitudes) and lower scores for external locus of control than obesity classes 1 and 2.

## 4. Discussion

This study aimed to explore maternal patterns of diet and PA behaviours, and GWG in a highly deprived population of women, and whether there were any differences in patterns between obesity classes. Overall, this population had suboptimal dietary intakes relating to low levels of consumption of oily fish, fruit and vegetables, wholemeal bread and unrefined breakfast cereals. EE tended to be from light-intensity PA, and habitual household/care and occupational modes of PA. The majority of women gained weight outside of the IoM guideline recommendations, including both inadequate and excessive GWG. The psychosocial analysis identified that this population of women had a negative body image, but paradoxically a high degree of self-efficacy and internal locus of control relating to their weight and related behaviours. When comparing obesity classes, there was a pattern in the data for women with class 3 obesity to have higher intake of bread, sugar-sweetened beverages, sweet snacks and red meat, EE from sedentary-intensity and inactive modes of PA, and a higher proportion of women with inadequate GWG below the IOM guideline recommendations. Women with class 3 obesity also had a lower overall EE, EE from light- or moderate-intensity PA and occupational mode of PA, lower odds of excessive GWG and a more negative body image. There was also some evidence that both diet and PA patterns change over the course of pregnancy when comparing samples 1 (data collection at approximately 20 weeks gestation) and 2 (data collection at approximately 36 weeks gestation).

This study adds to the scant literature on the habitual diet of UK pregnant women with obesity. The lack of adherence to dietary guidelines is consistent with previous reports and highlights the poor-quality diets consumed by pregnant women with obesity in the UK [31,47]. However, we have also reported a potential difference in dietary intake between obesity classes, with women in class 3 reporting more suboptimal dietary patterns compared to those in classes 1 and 2. This requires further research to better understand the relationship between dietary intake and severity of obesity to inform future guidelines for improving the health of pregnant women with obesity, especially considering the higher levels of inequality observed among women with class 3 obesity. Given the association between maternal diet in pregnancy and child weight from birth to adolescence [48], alongside the increased risk of childhood obesity development when mothers have an obese BMI before pregnancy [6], efforts to improve diet quality among pregnant women living with obesity has potential intergenerational benefits.

Patterns of self-reported PA in this study were similar to previously reported values using PPAQ [49]. As might be expected, the EE from occupation and transport related PA was lower in sample 2 (where data were collected at 36 weeks’ gestation) compared with sample 1 (data collected at 20 weeks’ gestation). The number of MET-hr/week spent in moderate intensity PA also decreased between samples 1 and 2. It is difficult to compare self-reported MET-hours per week to the UK Guidelines of 150 min of moderate intensity PA per week. It has been suggested that 16 MET h/week of PA equates to 41 min/day of walking [50]. However, similar to other studies [51] our data suggests a reduction in PA over the duration of pregnancy, particularly for women with class 3 obesity. This data reinforces the importance of supporting women to maintain both the amount and intensity of PA throughout pregnancy with the aim of preventing PA decline. Interestingly, only a small amount of total EE was expended carrying out sports activities, suggesting that most of the reported moderate intensity activity was not from formal exercise or sporting activities. There is evidence that some women are uncertain of appropriate exercise for pregnancy, with particular concerns relating to safety [52]. Women should be reassured that they can meet PA guidelines by carrying out habitual activities such as walking and actively playing with children, as well as appropriate sports- and exercise-based activities. Future research involving PA interventions in this population should focus on maintaining amount and intensity of habitual PA throughout pregnancy to help achieve national guideline recommendations, as well as exploring potential barriers to PA in pregnancy among women in different classes of obesity.

The data on GWG demonstrated a lack of adherence to the IOM-recommended GWG ranges for all women, with obesity class apparently influencing whether the GWG was inadequate or excessive. Other studies have demonstrated that GWG tends to decline, and the risk of weight loss increases, with increasing obesity class [53]. Our study provides novel data in the extent to which women with class 3 obesity appear to have a different overall patterns of GWG to women in classes 1 and 2. While there is a lack of conclusive mechanistic evidence as to why this pattern may have been observed, it may be related, in part, to resting energy expenditure (REE) which accounts for between 50 and 75% of total EE. In non-pregnant populations, REE is approximately 360 kcal/day higher among people living with obesity than for those without obesity, with differences observed according to obesity class: 240 kcal/day higher for class 1 obesity, and 540 kcal/day higher for class 3 obesity [54]. A study in pregnant women living with obesity also identified that increasing obesity class was significantly associated with increasing REE (obesity class 1: 1686 ± 39 kcal/day; class 2: 1775 ± 50 kcal/day; class 3: 2102 ± 58 kcal/day) [55]. A key contributor to REE is fat-free mass (FFM), although fat mass (FM) also contributes, and both are higher in people living with obesity than for those without obesity [54]. In pregnancy, increasing obesity class is significantly associated with both FFM (class 1: 50.4 ± 1.1 kg; class 2: 54.0 ± 1.5 kg; class 3: 60.7 ± 2.1 kg) and FM (class 1: 36.8 ± 1.0 kg; class 2: 44.8 ± 1.1 kg; class 3: 62.7 ± 2.9 kg) [55]. Further, REE increases over the course of pregnancy [56,57], and change in REE has been identified as being an important factor in determining GWG [58]. In a study with pregnant women (non-obese), change in REE correlated positively with changes in FFM and negatively with FM, and women with smaller increases in REE had higher GWG [58]. However, there is a lack of data exploring the extent to which change in REE differs between obesity classes. Based on evidence to date, it could be hypothesised that the lower GWG observed in the class 3 obesity group in GLOWING could be due to this population having a higher REE and increase in REE over the course of pregnancy, and a higher accrual of FFM than FM, compared with women with class 1 or 2 obesity. As such, the energy intake required for women with class 3 obesity to meet their REE demands, plus pregnancy-specific energy demands, and to gain excessive weight during pregnancy may be too high for some women, resulting in inadequate GWG. It is also important to note that our study applied the recommended GWG ranges for women with pre-pregnancy obesity (BMI ≥ 30.0 kg/m^2^) in order to explore adherence to guideline recommendations. This included a combination of first trimester GWG, and weekly GWG for the second and third trimesters. However, the IoM GWG guidelines do not differentiate between obesity classes in their recommendations for any of these trimester-specific GWG ranges. As discussed in the introduction to this paper, recent studies to date suggest that obesity class is an important factor in determining optimal GWG [23,24,25]. Collectively, these studies suggest that women in obesity classes 1–3 have different GWG requirements for prevention of adverse pregnancy outcomes, although there is disagreement on what these optimal ranges for each obesity class should be. The optimal GWG is reported by authors to be 5 kg to 9 kg [23], 0 kg [24], and 2 kg to <6 kg [25] for class 1 obesity; 1 kg to <5 kg [23], −4 kg [24], and “weight loss” or a GWG < 4 kg [25] for class 2 obesity; 0 kg [23], −5 kg [24] and 0 to <6 kg [25] for class 3 obesity. This provides further evidence for the need to treat obesity classes as separate populations in future research, guidelines and practice, as women living with obesity are not a homogeneous group.

In this study, we employed a novel method of determining adequacy of gestation-specific GWG informed by a published comparison of approaches [38]. Our approach used the latest weight measurement recorded in the second and third trimesters in order to optimise sample size (*n* = 90) due to the lack of consistency in gestational age at time of weight measurement, and the lack of preconception and delivery weight measurements which are required to apply the IoM total GWG guidelines [52]. As a sensitivity analysis, we compared these results to the GWG categorisations of a subgroup of women with weight recorded at 36 weeks’ (*n* = 51) to estimate the extent to which inconsistency and timing of measurement impacted on the results. We found that the results were highly comparable in both frequency and regression analyses which suggests that our approach of applying gestation-specific GWG ranges to weights recorded in the second or third trimester could be used to maximise sample size in future studies. We also performed multiple imputation to estimate weight at 36 weeks for all women (*n* = 163) and compared these with our complete case analyses. This showed generally similar trends in excessive GWG across obesity classes, but notably the effect size for excessive GWG reduced in those with class 3 vs. class 1 obesity when compared to complete cases analysis (OR = 0.34 vs. OR = 0.12). While this could be closer to the ‘true’ effect size, caution should be taken interpreting the results from the multiple imputation given the small sample size and the high proportion of missing weight data at 36 weeks (69%). While we were able to perform 20 iterations and include multiple important variables in the imputation, the limited complete case sample size meant we could not reasonably include all variables that might be important to predict 36 week weight (e.g., diet and PA data).

The psychosocial scales demonstrated negative body image, while also a high degree of internal locus of control (i.e., belief in their ability to control their own weight) and self-efficacy (i.e., confidence that they can control their weight and related behaviours). Most women also reported that they had successfully tried to lose weight in the past, which may be influencing the high degree of self-efficacy and internal locus of control. However, this pattern in the data may indicate internalised weight bias in this population, whereby women feel that the negative stereotypes about obesity causation (e.g., lack of will power, greed, laziness) apply to themselves [59]. In this context, the high internal locus of control may reflect that women feel they should have complete control of their weight and therefore having a high weight represents their failure in control (reflected in the negative body image scale). Others have identified that perceived weight stigma and internalised weight bias should be considered in the design and evaluation of interventions to improve mental health among people living with obesity [60]; however, there is a lack of research among pregnant populations.

### Strengths and Limitations

The data collection for the GLOWING pilot trial was extensive. The volume of data collected provides a rich dataset for analysis of patterns of diet, PA and GWG among a high risk and socio-economically disadvantaged population of women. However, as this was a secondary analysis of pilot trial data, the research was not designed or powered to detect statistically significant differences in the analysis we carried out. Due to this study being an exploratory analysis of a rich dataset on diet, PA and GWG, with data collection at different gestations and among women with different obesity classes, we have carried out multiple testing. The small sample size means that any statistically non-significant results does not necessarily mean there is no association between obesity classes and the outcomes explored, rather that this study is likely to be underpowered to detect a difference. As the aim of this study was to explore the patterns of diet and PA behaviours, and hereby identify areas for future research, we have not adjusted our findings for multiple testing [61], this is a similar approach taken by other analyses of trial data [62]. Therefore, the results of this study are descriptive, and we have primarily focused our discussion on the clinical significance relating to patterns in the data rather than statistical significance, with the purpose of identifying potential areas for future research. 

The data were collected from women representing a highly deprived population living in a high-income country. The socio-economic data were collected at both the area-level (i.e., IMD), and the individual-level (i.e., maternal education and employment). There was consistency in all of these indicators with a higher proportion of women in this sample living in areas of highest deprivation, having low levels of education and high rates of unemployment compared with national averages. There was also evidence that women living with class 3 obesity in this population had higher levels of some inequality measures than women living in the same geographic area with class 1 or 2 obesity, including measures relating to deprivation quintile, employment and education. However, this population was predominantly White, and women who did not speak English were excluded from this study, which will impact on how comparable our dataset is to other regions in the UK. There is a plethora of evidence which identifies that the social gradients in high income countries can result in significantly increased risks for women and their babies in the low socio-economic groups [63]. Further, all women in this study were at additional high risk due to their BMI being in the obese range, and have the greatest potential for benefit from interventions. We have very little existing evidence on patterns of diet, PA and GWG in populations of highly deprived women living with obesity, and this study provides a basis to inform future study direction. 

This exploratory study has identified multiple areas for future research, policy and practice. Women living with obesity and in areas with high levels of deprivation have a double burden of disease, as both obesity and deprivation increase risks of adverse pregnancy outcomes. This population of women require additional support to achieve guideline recommendations for diet, PA and GWG. Future research should explore the differences between obesity classes in both diet and PA behaviours and GWG to inform the development of guidelines; currently, diet and PA guidelines in the UK are not specific to women living with obesity and there are no UK GWG guidelines. The patterns in the psychosocial constructs and the potential role of internalised weight bias relating to maternal diet, PA and GWG should also be further explored. Further research into maternal diet, PA and GWG among obesity classes, with a particular focus on the needs of deprived populations, would inform the development of appropriate policies and provision of care to achieve the best health outcomes for women and their children.

## Figures and Tables

**Table 1:** GLOWING participant socio-demographic characteristics.

		Sample 1: 20 Weeks (*n* = 59)	Sample 2: 12 Weeks (*n* = 104)	Total Sample (*n* = 163)
Stage of pregnancy completing the questionnaire, weeks	Mean (SD)	20.8 (2.1)	12.8 (1.3)	NA
Booking BMI, kg/m^2^	Mean (SD)	34.5 (4.0)	37.2 (6.6)	36.2 (6.0)
	Class 1 *n* (%)	36 (61.0)	52 (50.0)	88 (54.0)
	Class 2 *n* (%)	15 (25.4)	27 (26.0)	42 (25.8)
	Class 3 *n* (%)	8 (13.6)	25 (24.0)	33 (20.2)
Maternal age, years	Mean (SD)	28.1 (4.9)	29.9 (5.4)	29.2 (5.3)
Number of pregnancies	Median (IQR)	2 (1 to 3)	2 (1 to 4)	2 (1 to 4)
Number of pregnancies beyond 24 weeks	Median (IQR)	1 (0 to 1)	1 (0 to 2)	1 (0 to 2)
Deprivation quintile, *n* (%)	Q1 (most deprived)	34 (57.6)	55 (52.9)	89 (54.6)
	Q2	16 (27.1)	20 (19.2)	36 (22.1)
	Q3	3 (5.1)	11 (10.6)	14 (8.6)
	Q4	5 (8.5)	10 (9.6)	15 (9.2)
	Q5 (least deprived)	1 (1.7)	7 (6.7)	8 (4.9)
	Missing	0	1 (1.0)	1 (0.6)
Usual employment, *n* (%)	Employed full time	22 (37.3)	42 (40.4)	64 (39.3)
	Employed part time	12 (20.3)	21 (20.2)	33 (20.2)
	Self-employed	3 (5.1)	3 (2.9)	6 (3.7)
	Unemployed	16 (27.1)	26 (25.5)	42 (25.8)
	Full-time student	3 (5.1)	2 (1.9)	5 (3.1)
	Unpaid carer for family/friend	0	4 (3.8)	4 (2.5)
	Other ^a^	1 (1.7)	4 (3.8)	5 (3.1)
	Missing	2 (3.4)	2 (1.9)	4 (2.5)
Has your employment changed during pregnancy? *n* (%)	No change	50 (84.7)	83 (79.8)	133 (81.6)
	I am on maternity leave	0	2 (1.9)	2 (1.2)
	I am on sick leave related to pregnancy	1 (1.7)	2 (1.9)	3 (1.8)
	I have reduced my working hours	3 (5.1)	3 (2.9)	6 (3.7)
	Other change ^b^	0	1 (1.0)	1 (0.6)
	Missing	5 (8.5)	13 (12.5)	18 (11.0)
Ethnic group, *n* (%) ^c^	White	54 (91.5)	99 (95.2)	153 (93.9)
	South Asian	2 (3.4)	2 (1.9)	4 (2.5)
	Mixed ethnic group	0	1 (1.0)	1 (0.6)
	Other ethnic group	2 (3.4)	1 (1.0)	3 (1.8)
	Prefer not to answer	0	1 (1.0)	1 (0.6)
	Missing	1 (1.7)	0	1 (0.6)
Education, *n* (%)	No formal qualifications	6 (10.2)	9 (8.7)	15 (9.2)
	GCSEs or equivalent	19 (32.2)	41 (39.4)	60 (36.8)
	A-levels or equivalent	17 (28.8)	27 (26.0)	44 (27.0)
	Bachelors degree or higher	9 (15.3)	15 (14.4)	24 (14.7)
	Other ^d^	1 (1.7)	11 (10.6)	12 (7.4)
	Missing	7 (11.9)	1 (1.0)	8 (4.9)
Relationship status, *n* (%) ^e^	Single	30 (50.8)	52 (50.0)	82 (50.3)
	Married	26 (44.1)	49 (47.1)	75 (46.0)
	Separated/divorced	1 (1.7)	1 (1.0)	2 (1.2)
	Widowed	1 (1.7)	1 (1.0)	2 (1.2)
	Missing	1 (1.7)	1 (1.0)	2 (1.2)

^a^ Other employment included paid carer, housewife, traineeship, and zero hours contract. ^b^ Other change to employment related to zero hour contract. ^c^ Ethnic group options were: White (White British, White Irish, Other White), South Asian (Bangladeshi, Indian, Pakistani, Other South Asian), Black (Black Caribbean, Black African, Other Black), mixed ethnic groups (White and Asian, White and Black African, White and Black Caribbean, Other Mixed), other ethnic groups (Chinese, Arabic, any other group), and prefer not to answer. ^d^ Other education included Apprenticeship, Diploma, General National Vocational Qualification (GNVQ), and National Vocational Qualification (NVQ). ^e^ Relationship status categories as defined in the questionnaire: single (including never married/civil partnership), married (including civil partnership), separated (still legally married/civil partnership) or divorced (including legally dissolved civil partnership), and widowed (including civil partnership). There was no relationship status option for women who were co-habiting who may have self-defined as being in any of the relationship status categories.

**Table 2:** Descriptive characteristics of the population for variables related to dietary behaviours.

		Sample 1: 20 Weeks Gestation (*n* = 59)	Sample 2: 36 Weeks Gestation (*n* = 39)	Total (*n* = 98)
Dietary preference *n* (%)	I eat both meat and fish	49 (83.1)	32 (82.1)	81 (82.7)
	I avoid meat but eat fish	2 (3.4)	0	2 (2.0)
	I avoid fish but eat meat	5 (8.5)	5 (12.8)	10 (10.2)
	I am a vegetarian and include dairy products and eggs in my diet	1 (1.7)	2 (5.1)	3 (3.1)
	Missing	2 (3.4)	0	2 (2.0)
Do you currently drink alcohol? *n* (%)	No	59 (100%)	37 (94.9)	96 (98.0)
	Yes	0	1 (2.6)	1 (1.0)
	Missing	0	1 (2.6)	1 (1.0)
	If yes, approximate number of units/week	NA	1 unit/week	1 unit/week
Do you currently smoke? *n* (%)	No, never smoked	29 (49.2)	22 (56.4)	51 (52.0)
	No, smoked in the past but never smoked during this pregnancy	17 (28.8)	13 (33.3)	30 (30.6)
	No, smoked previously in this pregnancy and not using any NRT	4 (6.8)	0	4 (4.1)
	No, smoked previously in this pregnancy and currently using NRT	1 (1.7)	0	1 (1.0)
	Currently smoke	6 (10.2)	4 (10.3)	10 (10.2)
	Missing	2 (3.4)	0	2 (2.0)
	If currently smoke—approx. *n* of cigarettes/day (range)	3 to 10/day	3 to 10/day	3 to 10/day
Do you currently suffer with nausea, *n* (%)	No	32 (54.2)	26 (66.7)	58 (59.2)
	Yes, daily	6 (10.2)	3 (7.7)	9 (9.2)
	Yes, less than daily	18 (30.5)	10 (25.6)	28 (28.6)
	Missing	3 (5.1)	0	3 (3.1)
Do you currently suffer with vomiting, *n* (%)	No	39 (66.1)	31 (79.5)	70 (71.4)
	Yes, daily	4 (6.8)	0	4 (4.1)
	Yes, less than daily	13 (22.0)	8 (20.5)	21 (21.4)
	Missing	3 (5.1)	0	3 (3.1)
Do you currently suffer with heartburn, *n* (%)	No	19 (32.2)	5 (12.8)	14 (14.3)
	Yes, daily	18 (30.5)	26 (66.7)	44 (44.9)
	Yes, less than daily	19 (32.2)	8 (20.5)	27 (27.6)
	Missing	3 (5.1)	0	3 (3.1)
If you answered yes to nausea, vomiting or heartburn (*n* = 77), has this changed the amount you are eating or drinking? *n* (%)	The same as before	20 (47.6)	14 (40.0)	34 (44.2)
	Less than before	15 (35.7)	16 (45.7)	31 (40.3)
	More than before	5 (11.9)	5 (14.3)	10 (13.0)
	Missing	2 (4.8)	0	2 (2.6)
Do you have any cravings to any foods or drinks, *n* (%)	No	47 (79.7)	24 (61.5)	71 (72.4)
	Yes ^a^	6 (10.2)	15 (38.5)	21 (21.4)
	Missing	6 (10.2)	0	6 (6.1)
Do you have any aversions to any foods or drinks, *n* (%)	No	43 (72.9)	27 (69.2)	70 (71.4)
	Yes ^b^	10 (16.9)	12 (30.8)	22 (22.4)
	Missing	6 (10.2)	0	6 (6.1)
Have you started to eat or drink anything different during this pregnancy that you did not have before you were pregnant? *n* (%)	No	43 (72.9)	27 (69.2)	70 (71.4)
	Yes ^c^	12 (20.3)	12 (30.8)	24 (24.5)
	Missing	4 (6.8)	0	4 (4.1)
Have you stopped eating or drinking anything different during this pregnancy that you used to have before you were pregnant? *n* (%)	No	33 (55.9)	16 (41.0)	49 (50.0)
	Yes ^d^	24 (40.7)	23 (59.0)	47 (48.0)
	Missing	2 (3.4)	0	2 (2.0)
Have you had an appointment with a dietitian? *n* (%)	No	NA ^e^	32 (82.1)	NA
	Yes		7 (17.9)	
Have you been diagnosed with GDM? *n* (%)	No	NA ^e^	34 (87.2)	NA
	Yes		5 (12.8)	

^a^ In the free-text responses, women described their cravings which were categorised into: sugar-sweetened products (*n* = 11: fizzy drinks (*n* = 4) and confectionary (*n* = 7)); fruit/fruit juice and vegetables (*n* = 8: citrus fruits (*n* = 3), fruit juice (*n* = 2) and vegetables (*n* = 3)); dairy products (*n* = 3); ice pops/cubes (*n* = 3); salty foods (*n* = 2); and rice (*n* = 1). ^b^ In the free-text responses, women described their aversions which were categorised into: meat (*n* = 9) and fish (*n* = 3); confectionary (*n* = 3); specific fruit and vegetables (*n* = 3); dairy (*n* = 2); hot drinks (*n* = 2); and spicy food (*n* = 2). ^c^ In the free-text responses, women described food and drink they had started eating during pregnancy which were categorised into: fruit/fruit juice and vegetables (*n* = 7); sugar sweetened products (*n* = 6: fizzy drinks (*n* = 4) and confectionary (*n* = 2)); water (*n* = 3); sandwiches (*n* = 2); milk (*n* = 1); and take away food (*n* = 1). ^d^ In the free-text responses, women described food and drink they had stopped eating during pregnancy which were categorised into: sugar sweetened drinks and confectionary (*n* = 12: chocolate specifically *n* = 7); caffeine/caffeinated drinks (*n* = 9); alcohol (*n* = 8); meat (or uncooked meat, *n* = 8); cheese (or certain types of cheese, *n* = 6); other dairy (*n* = 5); fish (or specific types of fish, *n* = 5); eggs (or specific ways of cooking eggs, *n* = 4); spicy food (*n* = 4); food not recommended in pregnancy (undefined, *n* = 2); nuts (*n* = 2); fruit juice (*n* = 2); and bread (*n* = 1). ^e^ The data collection time period for sample 1 was too early in the pregnancy for women to have seen a dietitian or had a diagnosis of GDM.

**Table 3:** Diet patterns for the total population, and stratified by obesity class.

	Total Population (*n* = 97)	Class 1 Obesity (*n* = 58)	Class 2 Obesity (*n* = 22)	Class 3 Obesity (*n* = 17)	*p*-Value *
Milk mL/day (median, IQR)	284 (142, 284)	284 (142, 568)	142 (142, 284)	284 (142, 426)	0.10
*Reduced fat (n, %)*	*67 (71.3%)*	*40 (72.7%)*	*16 (72.7%)*	*11 (64.7%)*	-
*Full fat (n, %)*	*20 (21.3%)*	*13 (23.6%)*	*3 (13.6%)*	*4 (23.5%)*	-
*None (n, %)*	*7 (7.4%)*	*2 (3.6%)*	*3 (13.6%)*	*2 (11.8%)*	-
Spread g/day (median, IQR)	7 (3, 11)	7 (3, 10 )	7 (4, 11)	10 (3, 18)	0.48
*Reduced fat (n, %)*	*45 (48.9%)*	*27 (48.2%)*	*10 (47.6%)*	*8 (53.3%)*	-
*Full fat (n, %)*	*35 (38.0%)*	*23 (41.1%)*	*7 (33.3%)*	*5 (33.3%)*	-
*None (n, %)*	*12 (13.0%)*	*6 (10.7%)*	*4 (19.0%)*	*2 (13.3%)*	-
Cheese g/day (median, IQR)	13 (4, 29)	13 (5, 26)	17 (7, 30)	17 (4, 47)	0.78
*Reduced fat (n, %)*	*4 (4.3%)*	*2 (3.6%)*	*1 (4.5%)*	*1 (6.3%)*	-
*Full fat (n, %)*	*60 (63.8%)*	*36 (64.3%)*	*14 (63.6%)*	*10 (62.5%)*	-
*None (n, %)*	*30 (31.9%)*	*18 (32.1%)*	*7 (31.8%)*	*5 (31.3%)*	-
Sugary drinks ml/day (median, IQR)	205 (56, 467)	208 (64, 559)	85 (14, 323)	247 (85, 623)	0.21
Fruit juice	28 (14, 85)	28 (14, 156)	85 (14, 85)	28 (0, 85)	0.56
Sugar-sweetened beverages	89 (4, 278)	91 (37, 287)	16 (0, 215)	190 (20, 500)	0.12
Starchy carbohydrate foods g/day (median, IQR)	269 (194, 315)	273 (185, 315)	300 (217, 341)	257 (238, 311)	0.65
Rice, pasta, noodles, potatoes	139 (88, 205)	125 (69, 199)	144 (103, 217)	142 (92, 172)	0.64
Takeaway and oven chips	21 (10, 39)	21 (10, 39)	30 (20, 67)	24 (15, 48)	0.49
Bread	41 (27, 62)	35 (21, 51)	51 (41, 72)	62 (41, 72)	0.01
*Wholemeal bread (n, %)*	*39 (41.9%)*	*24 (43.6%)*	*9 (40.9%)*	*6 (37.5%)*	-
*White bread (n, %)*	*49 (52.7%)*	*30 (54.5%)*	*10 (45.5%)*	*9 (56.3%)*	-
*No bread (n, %)*	*5 (5.4%)*	*1 (1.8%)*	*3 (13.6%)*	*1 (6.3%)*	-
Breakfast cereal	27 (16, 38)	23 (16, 38)	33 (27, 38)	22 (15, 34)	0.25
*Refined breakfast cereal (n, %)*	*23 (29.1%)*	*14 (28.0%)*	*4 (23.5%)*	*5 (41.7%)*	-
*Non-refined breakfast cereal (n, %)*	*36 (45.6%)*	*26 (52.0%)*	*7 (41.2%)*	*3 (25.0%)*	-
*No breakfast cereal (n, %)*	*20 (25.3%)*	*10 (20.0%)*	*6 (35.3%)*	*4 (33.3%)*	-
Fruits and vegetables g/day (median, IQR)	207 (106, 344)	218 (120, 331)	157 (77, 356)	203 (74, 310)	0.51
Vegetables	75 (38, 125)	75 (41, 144)	72 (33, 121)	77 (28, 126)	0.79
Fruits	122 (42, 194)	218 (120, 331)	157 (77, 356)	203 (74, 310)	0.56
Servings of fruit and vegetables per day ^a^	2.6	2.7	2.0	2.5	-
Snacks g/day (median, IQR)	90 (42, 146)	92 (44, 156)	55 (34, 123)	94 (52, 154)	0.45
Crisps and fried snacks	5 (0, 14)	2 (0, 14)	5 (0, 14)	5 (0, 14)	0.95
Sweet snacks	40 (19, 62)	45 (20, 64)	31 (13, 42)	51 (15, 86)	0.15
Yoghurt (g/day)	18 (0, 54)	36 (9, 54)	9 (0, 54)	18 (9, 54)	0.39
Meat and fish g/day (median, IQR)	143 (97, 180)	142 (91, 167)	168 (127, 203)	144 (122, 167)	0.33
Red meat	22 (11, 68)	22 (11, 68)	22 (22, 68)	68 (22, 68)	0.37
Processed meat and fish	27 (10, 53)	19 (10, 46)	37 (19, 55)	19 (10, 32)	0.10
Fish (all including processed)	17 (0, 41)	16 (0, 33)	25 (16, 50)	25 (12, 37)	0.19
Oily fish	0 (0, 9)	0 (0, 9)	4 (0, 17)	4 (0, 13)	0.28

* Statistical significance *p* < 0.05, *p*-value from Kruskal–Wallis test comparing obesity classes for the total population; - CHI^2^ test not possible due to *n* < 5 in some categories. ^a^ Servings of fruit and vegetables per day has been estimated based on the median total g/day for fruit and vegetables, with one serving being 80 g.

**Table 4:** Physical activity patterns for the total population overall and stratified by obesity class.

MET-hr/Week	Total Population (*n* = 93) ^a^	Class 1 Obesity (*n* = 55)	Class 2 Obesity (*n* = 21)	Class 3 Obesity (*n* = 17)	*p*-Value ^b^
Total (EE)	165.52 (128.12–249.20)	165.52 (128.93–247.48)	176.49 (134.97–266.62)	137.44 (117.51–271.00)	0.70
PA Intensity					
Sedentary-intensity PA	14.92 (7.35–28.00)	14.92 (7.35–18.90)	14.92 (4.56–29.40)	14.92 (7.35–28.92)	0.76
Light-intensity PA	111.38 (79.03–149.88)	116.36 (80.09–155.36)	125.94 (84.47–152.96)	86.80 (61.00–113.56)	0.03
Moderate-intensity PA	82.14 (45.72–134.81)	82.14 (45.70–125.74)	110.69 (67.40–134.81)	69.94 (22.05–143.28)	0.23
Vigorous-intensity PA	0.00 (0.00–0.78)	0.00 (0.00–0.78)	0.00 (0.00–0.39)	0.00 (0.00–0.39)	0.60
PA Mode					
Household/care PA	79.16 (41.40–135.51)	80.86 (40.80–147.01)	79.01 (36.36–135.53)	77.35 (46.91–123.93)	0.91
Occupational PA	71.57 (12.96–114.94)	71.57 (10.22–101.36)	71.57 (52.85–140.00)	30.80 (0.00–116.06)	0.43
Sport PA	1.14 (0.38–3.82)	1.14 (0.00–3.83)	1.600 (0.38–4.03)	1.02 (0.53–3.83)	0.59
Transport PA	15.96 (8.68–29.26)	17.36 (10.71–31.36)	10.71 (15.96–31.60)	12.11 (7.35–23.3)	0.32
Inactive PA	17.85 (8.86–29.26)	17.85 (8.86–28.00)	17.02 (12.18–31.74)	20.30 (10.85–30.44)	0.75

^a^ 93 out of 98 women had PPAQ data to estimate PA levels. ^b^ Statistical significance *p* < 0.05, *p*-value calculated via Kruskal–Wallis test comparing results for obesity classes for the total population. Abbreviations: METs = Metabolic Energy Equivalents, EE = energy expenditure, and PA = physical activity.

**Table 5:** Descriptive analysis of weight-related variables.

	Total (*n* = 163)	Class 1 Obesity (*n* = 88)	Class 2 Obesity (*n* = 42)	Class 3 Obesity (*n* = 33)	*p*-Value
Booking weight, kg (mean, SD)	99.5 (17.4)	88.3 (7.8)	98.6 (8.5)	123.9 (14.6)	<0.001
Have you ever tried to lose 10 lbs? *n* (%)					
Yes	141 (86.5)	75 (85.2)	36 (85.7)	30 (90.9)	-
No	17 (10.4)	11 (12.5)	6 (14.3)	0	
Missing	5 (3.1)	2 (2.3)	0	3 (9.1)	
If yes (*n* = 141), have your past weight loss attempts been successful? *N* (%)					
Very unsuccessful	20 (14.2)	11 (14.7)	4 (11.1)	5 (16.7)	0.365
Somewhat unsuccessful	21 (14.9)	6 (8.0)	8 (22.2)	7 (23.3)	
Somewhat successful	67 (47.5)	39 (52.0)	16 (44.4)	12 (40.0)	
Very successful	33 (23.4)	19 (25.3)	8 (22.2)	6 (20.0)	
Adequacy of gestation-specific GWG (*n* = 90 ^a^), *n* (%)					
Excessive (above IoM guidelines)	45 (50.0)	27 (61.4)	14 (63.6)	4 (16.7)	0.001
Not excessive (adequate or inadequate)	45 (50.0)	17 (38.6)	8 (36.4)	20 (83.3)	
Inadequate (below IOM guidelines)	34 (37.8)	12 (27.3)	8 (36.4)	14 (58.3)	
Adequate (within IoM guidelines)	11 (12.2)	5 (11.4)	0 (0.0)	6 (25.0)	

^a^ Data available for 90 women who either returned their third trimester questionnaires with the weight card, or had follow-up weight measurements in their routine medical records retrieved by audit.

**Table 6:** Psychosocial measures relating to weight and related behaviours, stratified by obesity class.

Scale	Class 1	Class 2	Class 3	*p*-Value
Body image before pregnancy, 4 questions (combined scale range 4 to 14) ^a^	6 (5, 7)	6 (4, 7)	5 (4, 6)	0.02
Internal locus of control, 2 questions (combined scale range 2 to 10) ^a^	9 (7, 10)	9 (7, 10)	9 (8, 10)	0.89
External locus of control, 2 questions (combined scale range 2 to 10) ^a^	7 (6, 9)	8 (6, 10)	6 (5, 9)	0.14
Self-efficacy, 8 questions (combined scale range 8 to 40) ^a^	31 (25, 36)	30 (25, 35)	31 (26, 35)	0.77
Attitudes to weight gain in pregnancy, 13 questions (combined scale range 13 to 52) ^b^	38.3 (8.1)	38.1 (7.6)	40.9 (6.9)	0.23
Feelings about motherhood, 7 questions (combined scale range 7 to 35) ^a^	27 (24, 31)	30 (25, 32)	27 (25, 30)	0.25
Feelings about career orientation, 13 questions (combined scale range 13 to 52) ^b^	36.9 (5.8)	36.7 (4.5)	36.5 (5.3)	0.94

^a^ not normally distributed, Kruskal–Wallis test, data are median and IQR. ^b^ normally distributed, ANNOVA test, data are mean and SD. Note: higher scores indicate more positive body image, attitudes to weight gain in pregnancy, feelings about motherhood, higher degree of self-efficacy and internal or external locus of control, and responses towards a greater preference for career orientation.

## Data Availability

The data presented in this study are available on request from the corresponding author. The data are not publicly available due to ethical approval restrictions and any further data sharing will be subject to necessary approvals being in place.

## Supplementary Materials

The following are available online at https://www.mdpi.com/article/10.3390/nu13061981/s1, Figure S1: GWG after booking for women in obesity classes 1, 2 and 3, compared to the IoM-recommended mean and range GWG for trimesters 2 and 3. Table S1: Exploration of differences in dietary behaviours between women in the GLOWING intervention and control clusters, in sample 1 and 2. Table S2: STROBE reporting guidelines checklist. Table S3: Conversion of FFQ items to dietary categories and subgroups. Table S4: Conversion of PPAQ to physical activity categories for intensity and mode of activity. Table S5: Calculations for determining adequacy of gestation-specific gestational weight gain (after booking) in the second and third trimester using IoM guidelines. Table S6: Comparison of the socio-demographics of women with missing or available questionnaire and weight data. Table S7: Comparison of the socio-demographics between obesity classes. Table S8: Comparison of dietary patterns stratified by the gestational age when the questionnaires were completed. Table S9: Comparison of PA patterns stratified by sample/gestational age when the questionnaires were completed. Table S10: Excessive gestational weight gain, comparing complete case analysis and multiple imputation approaches. Table S11: Results for psychosocial measures relating to weight and related behaviours, overall and stratified by gestational age at time of completing the questionnaire.
