# Supplementary material for: Diet, Physical Activity and Gestational Weight Gain Patterns among Pregnant Women Living with Obesity in the North East of England: The GLOWING Pilot Trial
Source: Nutrients. 2021 Jun 9;13(6):1981. doi: 10.3390/nu13061981 (PMC8227571; doi:10.3390/nu13061981)
Supplement: Supplementary file 1 [file nutrients-13-01981-s001.zip › nutrients-1204820-supplementary.pdf]

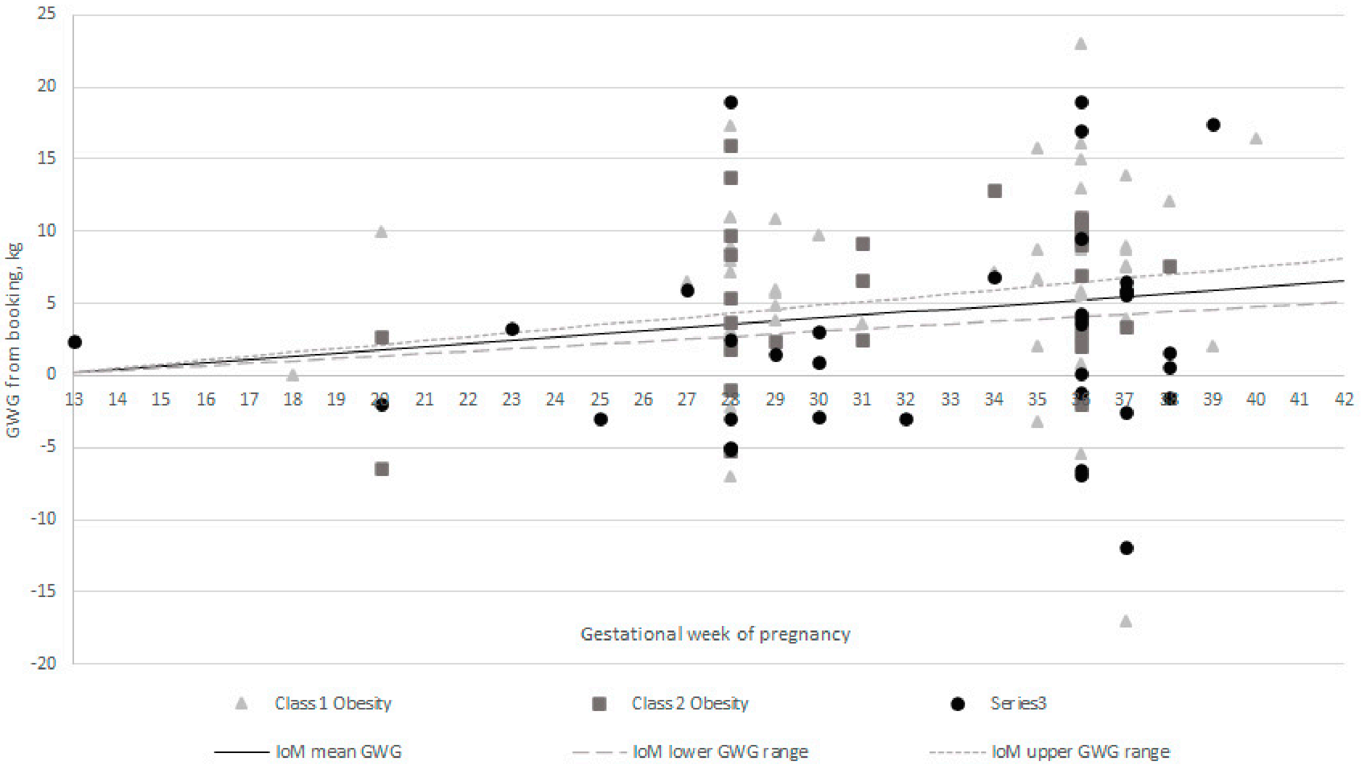

Figure S1. GWG after booking for women in obesity classes 1, 2 and 3, compared to the IoM recommended mean and range GWG for trimesters 2 and 3

S1 Table: Exploration of differences in dietary behaviours between women in the GLOWING intervention and control clusters, in samples 1 and 2

	**Sample 1 p-value[a]**	**Sample 2 p-value[b]**
**Dietary Outcomes**		
**Milk ml/day**	0.16	0.43
**Spread g/day**	0.91	0.39
**Cheese g/day**	0.29	0.07
**Sugary drinks ml/day**	0.65	0.16
Fruit juice	0.13	0.72
Sugar-sweetened beverages	0.86	0.06
**Starchy carbohydrates g/day**	0.051	0.58
Rice, pasta, noodles, potatoes	0.41	0.86
Takeaway & oven chips	0.13	0.70
Bread	0.19	0.87
Breakfast cereal	0.97	0.38
**Fruits and vegetables g/day**	0.41	0.18
Vegetables	**0.03**	0.26
Fruits	0.92	0.26
**Snacks g/day**	0.26	0.33
Crisps and fried snacks	0.16	0.07
Sweet snacks	0.55	0.74
Yoghurt (g/day)	0.11	0.33
**Meat and fish g/day**	0.17	0.72
Red meat	0.20	0.78
White meat	0.55	0.44
Processed meat	0.29	0.18
Processed fish	0.25	0.96
White fish	0.17	0.68

Oily fish	0.54	0.77
**PA Outcomes**		
**Total (EE)**	0.86	0.20
**Sedentary intensity PA**	0.38	0.42
**Light intensity PA**	0.95	0.35
**Moderate intensity PA**	0.47	0.16
**Vigorous intensity PA**	0.81	0.14
**Household/care PA**	0.22	0.83
**Occupational PA**	0.20	0.22
**Sport PA**	0.32	0.29
**Transport PA**	0.63	0.97
**Inactive PA**	0.45	0.42
**GWG Outcomes**		
**Excessive vs not excessive**	0.98	0.48

Note: Sample 1 data collection carried out prior to delivering the GLOWING intervention to midwives, data collection for diet and PA behaviours at approx. 20 weeks' gestation. Sample 2 data collection carried out after delivering the GLOWING intervention to midwives, data collection for diet and PA behaviours at approx. 36 weeks' gestation

[a] Statistical significance p<0.05, p-value from Mann-Whitney U test comparing intervention and control arms in sample 1

[b] Statistical significance p<0.05, p-value from Mann-Whitney U test comparing intervention and control arms in sample 2

S2 Table: STROBE reporting guidelines checklist

	Item No	Recommendation	Page No
**Title and abstract**	1	(*a*) Indicate the study's design with a commonly used term in the title or the abstract<br>(*b*) Provide in the abstract an informative and balanced summary of what was done and what was found	1
**Introduction**			
Background/rationale	2	Explain the scientific background and rationale for the investigation being reported	2
Objectives	3	State specific objectives, including any prespecified hypotheses	2
**Methods**			
Study design	4	Present key elements of study design early in the paper	2-3
Setting	5	Describe the setting, locations, and relevant dates, including periods of recruitment, exposure, follow-up, and data collection	2-3
Participants	6	(*a*) Give the eligibility criteria, and the sources and methods of selection of participants. Describe methods of follow-up<br>(*b*) For matched studies, give matching criteria and number of exposed and unexposed	3
Variables	7	Clearly define all outcomes, exposures, predictors, potential confounders, and effect modifiers. Give diagnostic criteria, if applicable	3-5
Data sources/ measurement	8*	For each variable of interest, give sources of data and details of methods of assessment (measurement). Describe comparability of assessment methods if there is more than one group	3-5
Bias	9	Describe any efforts to address potential sources of bias	5
Study size	10	Explain how the study size was arrived at	3
Quantitative variables	11	Explain how quantitative variables were handled in the analyses. If applicable, describe which groupings were chosen and why	5
Statistical methods	12	(*a*) Describe all statistical methods, including those used to control for confounding<br>(*b*) Describe any methods used to examine subgroups and interactions<br>(*c*) Explain how missing data were addressed<br>(*d*) If applicable, explain how loss to follow-up was addressed<br>(*e*) Describe any sensitivity analyses	3-5
**Results**			
Participants	13*	(a) Report numbers of individuals at each stage of study—eg numbers potentially eligible, examined for eligibility, confirmed eligible, included in the study, completing follow-up, and analysed<br>(b) Give reasons for non-participation at each stage<br>(c) Consider use of a flow diagram	5-6
Descriptive data	14*	(a) Give characteristics of study participants (eg demographic, clinical, social) and information on exposures and potential confounders<br>(b) Indicate number of participants with missing data for each variable of interest<br>(c) Summarise follow-up time (eg, average and total amount)	6
Outcome data	15*	Report numbers of outcome events or summary measures over time	7-14

Table S3: Conversion of FFQ items to dietary categories and sub-groups

High level category	Subgroup	FFQ items
**Milk**	Reduced fat, Full fat, None	Thinking about the last month, what type of milk did you use most often? (select one option)<br>None / Do not use milk, Full cream/ whole milk (blue top), Semi-skimmed milk (green top), Skimmed or 1% fat milk (red or orange top), Other, please specify:<br><br>Thinking about the last month, how much milk did you usually drink each day, including milk in tea, coffee and on cereals? (select one option)<br>None / Do not use milk, ¼ of a pint, ½ a pint, ¾ of a pint, 1 pint, 1 ¼ pints, 1 ½ pints, 1 ¾ pints, 2 pints or more
**Spread**	Reduced fat, Full fat, None	Thinking about the last month, what butter or spread did you usually use for spreading on bread, toast, rolls, crackers and using in mashed potatoes?<br>None / Do not use butter or spread for spreading or adding to mashed potatoes<br>OR Full brand name, Type / Description, Average use in last month, Amount per day (teaspoons)
**Cheese**	Reduced fat, Full fat, None	Thinking about the last month, what cheese did you usually eat?<br>None / Do not eat cheese<br>OR Type / Description, Average use in last month, Amount per day (matchbox- sized pieces/ teaspoons)
**Sugary drinks**	Fruit juice*	Fruit juice and juice drinks e.g. orange or mango juice, mixed fruit juice
	Sugar-sweetened beverages*	Squash (not diet or sugar free) e.g. Ribena<br>Fizzy drinks (not diet or sugar free) e.g. coke, lemonade<br>Tea or coffee with added sugar
**Starchy carbohydrates (Rice, pasta, noodles, potatoes, Takeaway & oven chips, Bread, Breakfast cereal, Cassava, fu fu, gari, kenkey, Plantain, yam or ackee)**	Rice, pasta, noodles, potatoes*	White or brown rice NOT basmati (plain)<br>White or brown basmati rice (plain)<br>Pilau rice, fried rice or jollof rice<br>Pasta/spaghetti (dried, fresh, stuffed)<br>Noodles (dried, fresh)<br>Potatoes ('new'/baby boiled)<br>Potatoes ('old' baked, boiled, roast or mashed)
	Takeaway & oven chips*	Takeaway chips, fries, potato products or curried potato (e.g. aloo)<br>Oven chips
	Bread (Wholemeal, White, None)	Thinking about the last month, what bread did you eat?<br>None / Do not eat bread<br>OR Full brand name, Type / Description, Average use in last month, Amount per day (slices or rolls)
	Breakfast cereal (Refined, Non-refined, None)	Thinking about the last month, what breakfast cereal (including muesli) and porridge did you eat?<br>None / Do not eat breakfast cereal or muesli<br>None / Do not eat porridge<br>OR Full brand name, Type / Description, Average use in last month
**Fruits and vegetables**	Vegetables (Fresh, frozen or tinned)*	Salad vegetables e.g. salad leaves/ lettuce, cucumber, celery, tomatoes<br>Onion<br>Broccoli, spring greens, kale, spinach, saag or other green vegetables e.g. okra, karela<br>Root vegetables e.g. carrots, radishes, beetroot, turnip, parsnips<br>Lentils, peas or beans (Dahl/dal/masoor), chana/chickpeas or vegetarian curry with pulses
	Fruits (Fresh, frozen or dried)*	Bananas<br>Citrus fruit e.g. oranges, satsumas, mandarins, grapefruit<br>Fresh fruit e.g. apples, pears, grapes, fruit salad, berries<br>Tropical fruits e.g. Mango, pineapple, lychee, melon, figs, dates<br>Dried fruit e.g. raisins, prunes
**Snacks**	Crisps and fried snacks*	Crisps, chevda (Bombay mix) or ganthia<br>South Asian fried snacks e.g. Dosa, Pakoras, Bhaji, bhaturas, paratha (stuffed)
	Sweet snacks*	Chocolate bar (milk/plain/white) e.g. Galaxy, Dairymilk, Twix, Mars<br>Cereal bar (oat, fruit, nut or seed)<br>Biscuits and cookies e.g. chocolate chip, digestive, custard cream, Penguin<br>Cakes and pastries e.g. doughnuts, Danish pastries, sponge cake, muffin, gateau<br>Sweets e.g. jelly sweets, chewy sweets, boiled sweets<br>South Asian sweets e.g. Kheer (rice pudding), kulfi, burfi, jelabi, gulab jaman, falooda, ladoo
	Yoghurt*	Yoghurt, fromage frais, lassi (sweet yoghurt drink) or raita
**Meat and fish (Red meat, White meat. Processed meat, Processed fish, White fish, Oily fish)**	Red meat*	Beef, pork, lamb, mutton, goat and other red meats (roast, steak, chops, slices, mince, curry)
	White meat*	Chicken, turkey (roast, slices, stew, mince)
	Processed meat*	Processed meat e.g. beef burger, sausages, kebab/doner<br>Meat products eg. pork pie, sausage roll, pasty, coated/breaded chicken
	Processed fish*	Fish in batter/crumbs, fish fingers, fishcakes
	White fish*	White fish, fresh or frozen e.g. cod, haddock, plaice, sole, halibut
	Oily fish*	Oily fish, fresh or canned e.g. mackerel, kippers, salmon, sardines, herring

* FFQ item responses: AVERAGE USE IN LAST MONTH (select one option per food or drink item) - Never (or less than once a month), 1-3 days a month, Once a week, 2-4 days per week, 5-6 days per week, Once a day, 2-3 a day, 4-5 a day, 6 or more a day

S4 Table: Conversion of PPAQ to physical activity categories for intensity and mode of activity

PA Categories	PPAQ question numbers	PPAQ item descriptions
**Vigorous intensity**	25, 26 and Qu 30, 31 (if open ended activities are >6.0 METs)	e.g., walking quickly up hills for fun or exercise; jogging
**Moderate intensity**	6, 8, 9, 10, 14, 19, 21, 23, 24, 27, 28, 29, 33, 35, 36 and 30, 31 (if open ended activities are ≥3.0 - ≤6.0 METs)	e.g., dressing, bathing or feeding a child while standing; playing with children while walking or running; carrying children; taking care of an older adult; playing with pets; mowing lawn using a walking mower; raking; gardening; walking quickly to go places; walking slowly for fun or exercise; walking more quickly for fun or exercise; prenatal exercise classes; swimming; dancing; standing or slowly walking at work while carrying things (heavier than 1 gallon milk jug); walking quickly at work while carrying things.
**Light intensity**	4, 5, 7, 11, 15, 16, 17, 18, 20, 22, 32, 34 and 30, 31 (if open ended activities are 1.5 - <3.0 METs)	e.g., preparing meals; dressing, bathing or feeding a child- while sitting; playing with children; light cleaning; shopping; heavy cleaning; mowing lawn while on a riding mower; walking slowly to go places; standing or slowly walking at work not carrying anything
**Sedentary**	12, 13 and 30, 31 (if open ended activities are <1.5 METs)	e.g., sitting and using a computer; sitting and reading or talking on the phone; driving or riding in a car; sitting at work or in class; watching TV or a video.
**Household / care**	4, 5, 6, 7, 8, 9, 10, 14, 15, 16, 17, 18, 19	e.g., preparing meals; dressing, bathing or feeding a child while sitting and standing; playing with children while sitting, standing, walking or running; carrying children; taking care of an older adult; light cleaning; shopping; heavy cleaning; mowing lawn while on riding mower or using a walking mower; raking and gardening.
**Occupation**	32, 33, 34, 35, 36	e.g., sitting at work or class; standing or slowly walking at work while carrying things or not (heavier than 1 gallon milk jug); walking quickly at work while carrying things or not
**Sport**	23, 24, 25, 26, 27, 28, 29, 30, 31	e.g., walking slowly or more quickly for fun and exercise; walking quickly up hills; jogging; prenatal exercise classes; swimming; dancing.
**Transport**	20, 21, 22	e.g., walking slowly or more quickly to go places such as to catch a bus or go to work, driving or riding in a car or bus
**Inactive**	11, 12, 13	e.g., sitting and using a computer (not for work), watching TV, reading or using the phone (not for work).

PA=physical activity; PPAQ=physical activity in pregnancy questionnaire

S5 Table: Calculations for determining adequacy of gestation-specific gestational weight gain (after booking) in the second and third trimester using IoM guidelines

Gestational week of pregnancy	Mean GWG, kg	Lower range GWG, kg	Upper range GWG, kg
13	0.22	0.17	0.27
14	0.44	0.34	0.54
15	0.66	0.51	0.81
16	0.88	0.68	1.08
17	1.1	0.85	1.35
18	1.32	1.02	1.62
19	1.54	1.19	1.89
20	1.76	1.36	2.16
21	1.98	1.53	2.43
22	2.2	1.7	2.7
23	2.42	1.87	2.97
24	2.64	2.04	3.24
25	2.86	2.21	3.51
26	3.08	2.38	3.78
27	3.3	2.55	4.05
28	3.52	2.72	4.32
29	3.74	2.89	4.59
30	3.96	3.06	4.86
31	4.18	3.23	5.13
32	4.4	3.4	5.4
33	4.62	3.57	5.67
34	4.84	3.74	5.94
35	5.06	3.91	6.21
36	5.28	4.08	6.48
37	5.5	4.25	6.75
38	5.72	4.42	7.02
39	5.94	4.59	7.29
40	6.16	4.76	7.56
41	6.38	4.93	7.83
42	6.6	5.1	8.1

GWG=gestational weight gain; IoM=Institute of Medicine

Note: The IoM total GWG recommendations for women with preconception obesity (BMI≥30.0 kg/m^2) are for 5-9kg. This total GWG recommendation assumes women will gain 0.5-2kg in the first trimester, and an incremental weight gain during the 2nd and 3rd trimester of 0.22kg/week (range 0.17-0.27kg). As GLOWING doesn't have a preconception weight, the booking weight was used, measured towards the end of the 1st trimester. Therefore, this weight measurement already incorporates the expected 1st trimester GWG element of the IoM recommended total GWG. The calculations for gestational age-specific GWG use the incremental recommendations for trimesters 2 and 3. Estimates of excessive, adequate, inadequate GWG are based on the upper and lower ranges of these gestation-specific GWGs.

S6 Table: Comparison of the socio-demographics of women with missing or available questionnaire and weight data

		Questionnaire missing (n=65)	Questionnaire returned (n=98)	p-value[a]	Follow up weight data missing (n=73)	Follow up weight data available (n=90)	p-value[b]
**Booking BMI, kg/m²** (mean, SD)		37.4 (7.1)	35.4 (5.0)	0.05	35.2 (5.4)	37.1 (6.26)	0.84
**Maternal age, years** (mean, SD)		29.0 (5.0)	29.0 (5.0)	0.20	28.0 (5.0)	30.0 (5.0)	0.07
**Number of pregnancies** (mean, SD)		2.9 (1.8)	2.6 (1.7)	0.44	2.7 (1.8)	2.7 (1.8)	0.95
**Number of pregnancies >24 weeks** (mean, SD)		1.45 (1.4)	1.08 (1.11)	0.10	1.34 (1.4)	1.14 (1.12)	0.08
**Deprivation quintile, n (%)**	Q1 (most deprived)	38 (59.4)	51 (52.0)	0.47	43 (59.7)	45 (50.6)	0.47
	Q2	11 (17.2)	25 (25.5)		16 (22.2)	20 (22.5)	
	Q3	7 (10.9)	7 (7.1)		6 (8.3)	8 (9.0)	
	Q4/5 (two least deprived)	8 (12.5)	15 (15.3)		7 (9.8)	16 (18.0)	
**Usual employment, n (%)**	Paid employment	36 (57.1)	67 (69.8)	0.10	44 (61.1)	59 (67.8)	0.38
	No paid employment	27 (42.9)	29 (30.2)		28 (38.9)	28 (32.2)	
**Ethnic group, n(%)**	White	62 (95.4)	91 (93.9)	-	72 (98.7)	81 (91.0)	-
	South Asian	1 (1.5)	3 (3.1)		0	4 (4.5)	
	Mixed Ethnic Group	1 (1.5)	0		0	1 (1.1)	
	Other Ethnic Group	0	3 (3.1)		1 (1.4)	2 (2.2)	
	Prefer not to Answer	1 (1.5)	0		0	1 (1.1)	
**Education, n (%)**	Up to high school leaver level	34 (52.3)	41 (45.6)	0.41	35 (50.0)	40 (47.1)	0.72
	Higer than high school level	31 (47.7)	49 (54.4)		35 (50.0)	45 (52.9)	
**Relationship status, n (%)**	Married	27 (41.5)	48 (50.0)	0.41	34 (47.2)	41 (46.1)	0.72
	Not married	38 (58.5)	48 (50.0)		38 (52.8)	48 (53.9)	

[a] Statistical significance p<0.05, p-value from T-test for comparing mean (SD) values and Chi-squared for comparing frequencies between women who returned their follow-up questionnaire and those that did not.
[b] Statistical significance p<0.05, p-value from T-test for comparing mean (SD) values and Chi-squared for comparing frequencies between women who had a gestational weight gain measurement recorded after their booking weight and those that did not.
Note: Collapsed groups for Chi-squared analysis to meet criteria of 5 per cell for deprivation, employment, education and relationship status.
- It was not possible to complete Chi-squared analyses for ethnic group due to limited number of non-White ethnic groups, meaning that the criteria for Chi-squared test were not fulfilled.

S7 Table: Comparison of the socio-demographics between obesity classes

		Class 1 Obesity (n=58)	Class 2 Obesity (n=22)	Class 3 Obesity (n=17)
**Maternal age, years**	Mean (SD)	29 (5)	30 (5)	31 (5)
**Number of pregnancies**	Median (IQR)	2 (1, 4)	2 (2, 3)	3 (1, 4)
**Number of pregnancies beyond 24 weeks** Median (IQR)		1 (0, 2)	1 (0, 2)	1 (0, 2)
**Deprivation quintile, n (%)**	Q1 (most deprived)	46 (52.9)	23 (54.8)	19 (59.4)
	Q2	20 (23.0)	9 (21.4)	7 (21.9)
	Q3	8 (9.2)	2 (4.8)	4 (12.5)
	Q4	9 (10.3)	5 (11.9)	1 (3.1)
	Q5 (least deprived)	4 (4.6)	3 (7.1)	1 (3.1)
**Usual employment, n (%)**	Employed Full Time	39 (44.3)	15 (38.5)	10 (31.3)
	Employed Part Time	16 (18.2)	10 (25.6)	7 (21.9)
	Self Employed	3 (3.4)	3 (7.7)	0
	Unemployed	22 (25.0)	7 (17.9)	13 (40.6)
	Full Time Student	3 (3.4)	1 (2.6)	1 (3.1)
	Unpaid Carer for Family/Friend	3 (3.4)	1 (2.6)	0
	Other	2 (2.3)	2 (5.1)	1 (3.1)
**Ethnic group, n (%)**	White	82 (93.2)	39 (95.1)	32 (97.0)
	South Asian	3 (3.4)	1 (2.4)	0
	Mixed Ethnic Group	1 (1.1)	0	0
	Other Ethnic Group	2 (2.3)	0	1 (3.0)
	Prefer not to Answer	0	1 (2.4)	0
**Education, n (%)**	No formal qualifications	5 (6.0)	4 (10.0)	6 (19.4)
	GCSEs or equivalent	34 (40.5)	10 (25.0)	16 (51.6)
	A-levels or equivalent	30 (35.7)	11 (27.5)	3 (9.7)
	Bachelors degree or higher	10 (11.9)	10 (25.0)	4 (12.9)
	Other	5 (6.0)	5 (12.5)	2 (6.5)
**Relationship status, n (%)**	Single	44 (50.6)	21 (50.0)	17 (53.1)
	Married	43 (49.4)	20 (47.6)	12 (37.5)
	Separated/divorced	0	0	2 (6.2)
	Widowed	0	1 (2.4)	1 (3.1)

S8 Table: Comparison of dietary patterns stratified by the gestational age when the questionnaires were completed

Median (IQR) unless otherwise specified	Sample	Total population (n=97)	p-value[a]	Class 1 Obesity (n=58)	Class 2 Obesity (n=22)	Class 3 Obesity (n=17)	p-value[b]
**Milk ml/day**	1: 20 weeks	142 (142, 284)	0.30	284 (142, 284)	142 (142, 142)	213 (71, 426)	0.09
	2: 36 weeks	284 (142, 426)		284 (142, 568)	284 (142 284	284 (142, 426)	0.77
Reduced fat (n, %)	*1: 20 weeks*	*40 (71.4%)*		*24 (72.7%)*	*11 (73.3%)*	*5 (62.5%)*	-
	2: 36 weeks	*27 (71.1%)*		*16 (72.7%)*	*5 (71.4%)*	*6 (66.7%)*	
Full fat (n, %)	*1: 20 weeks*	*9 (16.1%)*		*7 (21.2%)*	*1 (6.7%)*	*1 (12.5%)*	-
	2: 36 weeks	*11 (28.9%)*		*6 (27.3%)*	*3 (33.3%)*	*2 (28.6%)*	
None (n, %)	*1: 20 weeks*	*7 (12.5%)*		*2 (6.1%)*	*3 (20.0%)*	*2 (25.0%)*	-
	2: 36 weeks	*0*		*0*	*0*	*0*	
**Spread g/day**	1: 20 weeks	6 (3, 11)	0.48	5 (3, 11)	6 (3, 11)	7 (3, 18)	0.92
	2: 36 weeks	9 (4, 10)		7 (3, 10)	10 (5, 11)	10 (4, 18)	0.56
Reduced fat (n, %)	*1: 20 weeks*	*28 (50.9%)*		*17 (50.0%)*	*7 (50.0%)*	*4 (57.1%)*	-
	2: 36 weeks	*17 (45.9%)*		*10 (45.5%)*	*3 (42.9%)*	*4 (50.0%)*	
Full fat (n, %)	*1: 20 weeks*	*17 (30.9%)*		*13 (38.2%)*	*3 (21.4%)*	*1 (14.3%)*	-
	2: 36 weeks	*18 (48.6%)*		*10 (45.5%)*	*4 (57.1%)*	*4 (50.0%)*	
None (n, %)	*1: 20 weeks*	*10 (18.2%)*		*4 (11.8%)*	*4 (28.6%)*	*2 (28.6%)*	-
	2: 36 weeks	*2 (5.4%)*		*2 (9.1%)*	*0*	*0*	
**Cheese g/day**	1: 20 weeks	15 (8, 28)	0.36	14 (8, 28)	9 (4, 20)	26 (17, 69)	0.27
	2: 36 weeks	9 (4, 31)		8 (4, 18)	25 (9, 34)	9 (3, 42)	0.36
Reduced fat (n, %)	*1: 20 weeks*	*1 (1.8%)*		*0*	*1 (6.7%)*	*0*	-
	2: 36 weeks	*3 (7.9%)*		*2 (9.1)*	*0*	*1 (11.1%*	
Full fat (n, %)	*1: 20 weeks*	*33 (58.9%)*		*22 (64.7%)*	*8 (53.3%)*	*3 (42.9%)*	-
	2: 36 weeks	*27 (71.1%)*		*14 (63.6%*	*6 (85.7%)*	*7 (77.8%)*	
None (n, %)	*1: 20 weeks*	*22 (39.3%)*		*12 (35.3%)*	*6 (40.0%)*	*4 (57.1%)*	-
	2: 36 weeks	*8 (21.1%)*		*6 (27.3%)*	*1 (14.3%)*	*1 (11.1%)*	
**Sugary drinks ml/day**	1: 20 weeks	162 (73, 323)	0.78	154 (73, 289)	193 (50, 328)	155 (85, 254)	0.98
	2: 36 weeks	226 (28, 640)		235 (57, 709)	28 (0, 85)	469 (130, 788)	0.06
Fruit juice	1: 20 weeks	85 (21, 156)	0.04	85 (14, 156)	85 (28, 156)	85 (28, 85)	0.81
	2: 36 weeks	28 (0, 85)		28 (14, 85)	28 (0, 85)	0 (0, 85)	0.43
Sugar-sweetened beverages	1: 20 weeks	89 (18, 235)	0.65	89 (35, 223)	108 (5, 243	94 (0, 227)	0.90
	2: 36 weeks	93 (0, 510)		137 (37, 520)	0 (0, 0)	393 (87, 705)	0.01
**Starchy carbohydrates g/day**	1: 20 weeks	238 (173, 300)	0.01	229 (167, 299)	200 (173 217	282 (246, 323)	0.30
	2: 36 weeks	292 (262, 344)		292 (264, 344)	315 (287 384	257 (187, 265)	0.11
Rice, pasta, noodles, potatoes	1: 20 weeks	132 (89, 217)	0.82	124 (69, 219)	126 (89 217	172 (94, 195)	0.80
	2: 36 weeks	143 (88, 174)		133 (70, 178)	147 (141 222	93 (67, 148)	0.19
Takeaway & oven chips	1: 20 weeks	21 (20, 39)	0.32	25 (19, 39)	25 (20 67	20 (20, 56)	0.92
	2: 36 weeks	21 (10, 39)		21 (10, 29)	39 (20 67	29 (10, 39)	0.26
Bread	1: 20 weeks	41 (21, 51)	0.02	31 (21, 51)	51 (41 62	51 (33, 72)	0.02
	2: 36 weeks	51 (31, 72)		41 (31, 69)	51 (31 72	72 (54, 99)	0.19
Wholemeal bread (n, %)	*1: 20 weeks*	*21 (37.5%)*		*14 (42.4%)*	*5 (33.3%)*	*2 (25.0%)*	-
	2: 36 weeks	*18 (48.6%)*		*10 (45.5%*	*4 (57.1%)*	*4 (50.0%)*	
White bread (n, %)	*1: 20 weeks*	*32 (57.1%)*		*19 (57.6%)*	*7 (46.7%)*	*6 (75.0%)*	-
	2: 36 weeks	*17 (45.9%)*		*11 (50.0%)*	*3 (42.9%)*	*3 (37.5%)*	
No bread (n, %)	*1: 20 weeks*	*3 (5.4%)*		*0*	*3 (20.0%)*	*0*	-
	2: 36 weeks	*2 (5.4%)*		*1 (4.5%)*	*0*	*1 (12.5%)*	
Breakfast cereal	20 weeks	21 (14, 33)	0.01	18 (14, 33)	27 (16, 35)	24 (14, 34)	0.88
	36 weeks	28 (22, 70)		33 (20, 88)	38 (28, 84)	22 (15, 33)	0.30
Refined cereal (n, %)	*1: 20 weeks*	*13 (30.2%)*		*9 (32.1%)*	*2 (20.0%)*	*2 (40.0%)*	-
	2: 36 weeks	*10 (27.8%)*		*5 (22.7%)*	*2 (28.6%)*	*3 (42.9%)*	
Non-refined cereal (n, %)	*1: 20 weeks*	*19 (44.2%)*		*15 (53.6%)*	*2 (20.0%)*	*2 (40.0%)*	-
	2: 36 weeks	*17 (47.2%)*		*11 (50.0%)*	*5 (71.4%)*	*1 (14.3%)*	
No cereal (n, %)	*1: 20 weeks*	*11 (25.6%)*		*4 (14.3%)*	*6 (60.0%)*	*1 (20.0%)*	-
	2: 36 weeks	*9 (25.0%)*		*6 (27.3%)*	*0*	*3 (42.9%)*	
**Fruits and vegetables g/day**	1: 20 weeks	195 (106, 360)	0.90	187 (109, 322	206 (84, 356)	275 (130, 413)	0.78
	2: 36 weeks	218 (103, 284)		245 (150, 331	92 (57, 333)	196 (55, 243)	0.12
Vegetables	1: 20 weeks	66 (35, 121)	0.29	62 (38, 122)	71 (24, 121)	72 (36, 124)	0.94
	2: 36 weeks	78 (39, 144)		78 (58, 145)	78 (36, 197)	80 (28, 126)	0.88
Fruits	1: 20 weeks	139 (48, 227)	0.29	116 (59, 187)	136 (42, 242)	214 (81, 308)	0.54
	2: 36 weeks	113 (42, 160)		139 (96, 194)	38 (21, 135)	76 (26, 117)	0.03
**Snacks g/day**	1: 20 weeks	81 (33, 128)	0.06	107 (35, 151)	52 (28, 88)	57 (33, 81)	0.09
	2: 36 weeks	105 (53, 165)		78 (51, 160)	135 (52, 207)	136 (94, 185)	0.46
Crisps/fried snacks	1: 20 weeks	2 (0, 14)	0.28	3 (0, 22)	2 (0, 14)	1 (0, 5)	0.44
	2: 36 weeks	5 (2, 14)		2 (0, 14)	5 (2, 33)	14 (3, 14)	0.21

Sweet snacks	1: 20 weeks	34 (16, 53)	0.02	45 (20, 65)	22 (11, 38)	23 (15, 50)	0.05
	2: 36 weeks	47 (28, 86)		45 (27, 64)	43 (28, 174)	74 (34, 114)	0.55
Yoghurt	1: 20 weeks	18 (0, 54)	0.21	36 (0, 54)	9 (0, 54)	13 (0, 18)	0.30
	2: 36 weeks	54 (9, 54)		36 (9, 54)	54 (0, 99)	54 (9, 54)	0.97
**Meat and fish g/day**	1: 20 weeks	143 (109, 168)	0.75	143 (105, 168)	149 (127, 202)	137 (124, 151)	0.84
	2: 36 weeks	144 (88, 194)		122 (57, 158)	198 (84, 327)	157 (122, 191)	0.18
Red meat	1: 20 weeks	22 (11, 68)	0.92	22 (11, 68)	22 (11, 68)	68 (22, 68)	0.36
	2: 36 weeks	22 (11, 68)		22 (0, 68)	68 (22, 125)	45 (17, 68)	0.12
Processed meat/fish	1: 20 weeks	27 (18, 55)	0.63	27 (18, 55)	41 (19, 59)	18 (10, 23)	0.03
	2: 36 weeks	27 (10, 38)		19 (9, 37)	37 (10, 55)	32 (14, 66)	0.38
Fish (incl processed)	1: 20 weeks	18 (7, 41)	0.21	16 (7, 34)	25 (16, 50)	25 (12, 38)	0.38
	2: 36 weeks	16 (0, 34)		8 (0, 33)	25 (0, 50)	24 (8, 37)	0.49
Oily fish	1: 20 weeks	0 (0, 17)	0.09	0 (0, 9)	9 (0, 17)	9 (4, 13)	0.24
	2: 36 weeks	0 (0, 9)		0 (0, 0)	0 (0, 9)	0 (0, 9)	0.76

[a] Statistical significance $p<0.05$, p-value from Mann-Whitney U test comparing sample 1 (diet data collection at approx. 20 weeks) and sample 2 (diet data collection at approx. 36 weeks)

[b] Statistical significance $p<0.05$, p-value from Kruskal Wallis test comparing obesity classes for sample 1 (diet data collection at approx. 20 weeks), and comparing obesity classes for sample 2 (diet data collection at approx. 36 weeks)

- Chi^2 test not possible due to $n<5$ in some categories

S9 Table: Comparison of PA patterns stratified by sample / gestational age when the questionnaires were completed

MET-hr/week	Sample	Total population (n=93)[a]	p-value [b]	Class 1 Obesity (n=55)	Class 2 Obesity (n=21)	Class 3 Obesity (n=17)	p-value [c]
Total (EE)	1: 20 weeks	172.11 (128.55-269.93)	0.11	166.41 (123.73-279.71)	186.20 (150.53-259.19)	167.71 (128.67-287.83)	0.75
	2: 36 weeks	155.19 (106.81-217.27)		163.48 (131.72-223.53)	155.99 (94.31-289.93)	131.60 (86.83-238.19)	0.60
Sedentary intensity	1: 20 weeks	14.92 (7.14-18.90)	0.39	14.92 (7.35-18.90)	11.14 (4.42-29.96)	7.35 (6.53-17.85)	0.65
	2: 36 weeks	17.85 (7.35-28.92)		16.39 (4.19-28.00)	14.92 (7.35-29.40)	22.92 (12.34-29.04)	0.61
Light intensity	1: 20 weeks	115.58 (90.48-154.31)	0.05	119.91 (92.68-156.38)	127.74 (85.26-150.42)	113.05 (68.95-114.79)	0.56
	2: 36 weeks	100.80 (64.47-144.56)		110.03 (79.24-157.25)	115.62 (62.79-161.35)	68.10 (53.49-103.66)	0.12
Moderate intensity	1: 20 weeks	91.43 (66.90-151.59)	0.04	86.38 (56.45-156.92)	110.92 (82.83-133.40)	89.60 (45.74-155.07)	0.48
	2: 36 weeks	76.68 (25.58-121.33)		81.73 (27.15-120.99)	79.90 (12.86-154.18)	54.52 (8.97-93.31)	0.41
Vigorous intensity	1: 20 weeks	0.00 (0.00-0.78)	0.81	0.00 (0.00-0.78)	0.00 (0.00-0.78)	0.00 (0.00-0.00)	0.53
	2: 36 weeks	0.00 (0.00-0.78)		0.00 (0.00-0.78)	0.00 (0.00-0.00)	0.00 (0.00-0.78)	0.12
Household / care	1: 20 weeks	88.64 (39.95-147.52)	0.49	90.30 (39.82-147.81)	83.63 (29.47-160.82)	103.25 (77.35-141.46)	0.69
	2: 36 weeks	70.98 (42.70-106.95)		75.98 (42.40-113.46)	65.80 (52.01-116.42)	52.60 (37.55-102.42)	0.51
Occupation	1: 20 weeks	77.24 (38.82-137.34)	0.02	79.55 (27.38-130.20)	78.57 (67.20-137.34)	74.90 (0.00-166.82)	0.76
	2: 36 weeks	33.32 (0.00-98.00)		37.31 (1.89-98.63)	69.05 (0.00-145.32)	21.57 (0.00-102.69)	0.93
Sport	1: 20 weeks	1.12 (0.29-4.68)	0.50	0.72 (0.00-4.25)	1.66 (0.38-6.16)	1.60 (0.00-6.20)	0.30
	2: 36 weeks	1.16 (0.74-3.77)		1.16 (0.65-4.40)	0.94 (0.30-3.38)	0.97 (0.75-2.73)	0.78
Transport	1: 20 weeks	16.03 (12.11-36.75)	0.04	17.36 (12.60-38.50)	16.73 (13.06-43.31)	12.11 (7.35-29.26)	0.44
	2: 36 weeks	13.37 (6.72-24.01)		17.60 (7.19-28.00)	10.71 (6.72-26.46)	11.69 (3.04-22.96)	0.64
Inactive	1: 20 weeks	17.16 (8.66-25.46)	0.33	17.85 (8.86-22.28)	17.16 (9.59-31.69)	13.65 (7.35-30.45)	0.78
	2: 36 weeks	24.15 (13.65-30.44)		20.29 (5.33-28.00)	16.44 (13.65-42.00)	26.08 (15.74-33.33)	0.53

[a] 93 out of 98 women had PPAQ data to estimate PA levels; 54 out of 59 women in sample 1 and 39 out of 39 in sample 2.
Sample size for sample 1 (n=54): Class 1 obesity (n=33), Class 2 obesity (n=14), Class 3 obesity (n=7)
Sample size for sample 2 (n=39): Class 1 obesity (n=22), Class 2 obesity (n=7), Class 3 obesity (n=10)
[b] Statistical significance p<0.05, p-value from Mann-Whitney U test comparing sample 1 (PA data collection at approx. 20 weeks) and sample 2 (PA data collection at approx. 36 weeks)
[c] Statistical significance p<0.05, p-value from Kruskall-Wallis test comparing obesity classes for sample 1 (PA data collection at approx. 20 weeks), and comparing obesity classes for sample 2 (PA data collection at approx. 36 weeks)
Abbreviations: METs = Metabolic Energy Equivalents, EE = Energy Expenditure, PA = physical activity

S10 Table: Excessive gestational weight gain, comparing complete case analysis and multiple imputation approaches

	% Excessive GWG			OR and AOR (95% CI)		
	Class 1 obesity	Class 2 obesity	Class 3 obesity	Class 1 obesity	Class 2 obesity	Class 3 obesity
**CCA for excessive GWG using weights measured at any gestation (n=90)**	61.4%	63.6%	16.7%	1 (reference)	OR 1.10 (0.38, 3.18)<br>AOR 1.04 (0.36, 3.06)	OR 0.13 (0.37, 0.43)<br>AOR 0.13 (0.04, 0.45)
**CCA for excessive GWG using weights measured at 36 weeks gestation (n=51)**	63.6%	66.7%	17.6%	1 (reference)	OR 1.14 (0.26, 5.03)<br>AOR 0.99 (0.21, 4.60)	OR 0.12 (0.03, 0.56)<br>AOR 0.12 (0.03, 0.58)
**MI for excessive GWG at 36 weeks gestation (n=163)**	55.5%	50.9%	29.9 %	1 (reference)	OR 0.82 (0.26, 2.55)<br>AOR 0.84 (0.26, 2.67)	OR 0.33 (0.08, 1.33)<br>AOR 0.34 (0.08, 1.43)

CCA=complete case analysis; GWG=gestational weight gain; OR=odds ratio; AOR=adjusted odds ratio (inlcuding intervention arm and time point pre- or post-intervention sample); CI=confidence interval; MI=multiple imputation; Class 1 obesity BMI 30.0 to 34.9kg/m^2; Class 2 obesity BMI 35.0 to 39.9kg/m^2; Class 3 obesity BMI ≥40.0kg/m^2

S11 Table: Results for psychosocial measures relating to weight and related behaviours, overall and stratified by gestational age at time of completing the questionnaire (Table adapted from Fealey et al 2020)

**Weight Locus of Control (LOC)** (Likert scale 1 to 5, underlined scores measure internal LOC)	**Strongly Agree / Agree (1-2)**	**Neither Agree nor Disagree (3)**	**Strongly Disagree / Disagree (4-5)**	**Missing Responses (n)**	**p-value[a]**
Q1 *Whether my weight change is up to me (Internal LOC)	**79.4**	13.1	7.5	3	0.46
Q2 *If I eat right, and get enough exercise and rest, I can control my weight the way I want. (Internal LOC)	**81.9**	10.0	8.1	3	0.61
Q3 Being the right weight is mainly good luck (External LOC)	19.4	22.5	**58.1**	3	0.47
Q4 No matter what I try to do, if I gain or lose weight, or stay the same, it is just going to happen. (External LOC)	22.5	33.1	44.4	3	0.20
**Self-Efficacy** (Likert scale 1 to 5, underlined scores measure greater self-efficacy). How sure are you that you can:	**Very Sure / Sure** (1-2)	**Neither sure nor Unsure** (3)	**Very Unsure / Unsure** (4-5)		
Q5 *Fit into your regular clothes	**62.7**	12.0	25.3	5	0.39
Q6 *Take off any extra weight you gain	**61.9**	16.3	21.9	3	**0.03**
Q7 *Get back into shape	**65.6**	13.1	21.3	3	0.23
Q8 *Eat balanced meals	**79.4**	11.9	8.7	3	0.17
Q9 *Eat foods that are good for you and avoid foods that are not	**72.5**	8.8	18.8	3	0.96
Q10 *Eat foods that are good for you even when family or social life takes a lot of your time.	**65.8**	14.6	19.6	5	0.21
Q11 *Get regular exercise	**70.6**	12.5	16.9	3	0.24
Q12 *Get regular exercise even when family or social life takes a lot of your time.	**60.6**	18.1	21.3	3	0.17
**Attitudes towards weight gain (**Likert scale 1 to 5, underlined scores measure positive attitudes towards weight gain)	**Strongly Agree / Agree** (1-2)	**Neither Agree nor Disagree** (3)	**Strongly Disagree / Disagree** (4-5)		
Q13 *The weight I gain during pregnancy makes me feel ugly	43.4	26.4	30.2	4	0.31
Q14 *I worry that I may get fat during this pregnancy	**52.8**	18.2	28.9	4	0.36
Q15 *I am embarrassed at how big I've gotten during this pregnancy	29.6	29.6	40.9	4	0.69
Q16 *I'm embarrassed whenever the nurse weighs me	42.5	20.0	37.5	3	0.65
Q17 *I am trying to keep my weight down, so I don't look pregnant	9.4	22.5	**68.1**	3	0.63
Q18 I would like to gain between 25 and 30 pounds during this pregnancy	20.8	48.4	30.8	4	0.82
Q19 I would gain 40 pounds if it meant a healthier baby	44.0	28.9	27.0	4	0.87
Q20 *I will feel badly if I gain more than 20 pounds during this pregnancy	26.3	38.1	35.6	3	0.32
Q21 I like being able to gain weight for a change	6.9	22.5	**70.6**	3	0.77
Q22 As long as I'm eating a well-balanced diet, I don't care how much I gain during this pregnancy.	48.4	23.6	28.0	2	0.88
Q23 *I'm sure I will be able to fully control the amount of weight I will gain during this pregnancy	29.8	33.5	36.6	2	0.053
Q24 You can't totally control the amount of weight you gain when you are pregnant	**57.8**	23.6	18.6	2	0.66
Q25 *I feel that women have to be very careful about getting fat during pregnancy.	40.4	36.0	23.6	2	0.24
**Body Image: satisfaction** (Likert scale 1 to 4, underlined scores measure preference towards satisfaction with body weight/ shape)	**Very Satisfied / Satisfied**	**Very Dissatisfied / Dissatisfied**	-		
Q26 *How satisfied are you with your current shape?	27.0	**73.0**	-	4	0.11
Q27 *How satisfied are you with your current weight?	18.6	**81.4**	-	2	0.18

**Body Image: perception** (Likert scale 1 to 3, underlined scores measre preference towards a positive body weight/shape perception)	**Too Heavy (weight) / Big (shape)**	**About Right**	**Too Light (weight) / Thin (shape)**		
Q28 Do you consider your current weight to be…	**88.7**	11.3	0	4	0.42
Q29 Do you consider your current body shape to be…	**86.9**	13.1	0	3	0.22
**Measures of Feelings About Motherhood** (Likert scale 1 to 5, underlined scores measure positive feelings)	**Strongly Agree / Agree** (1-2)	**Neither Agree nor Disagree** (3)	**Strongly Disagree / Disagree** (4-5)		
Q30 Having a baby brings a lot of stress into a woman's life.	46.0	31.7	22.4	2	0.97
Q31 I'm not sure how I will manage after I have the baby	10.6	19.3	**70.2**	2	0.90
Q32 I am afraid I will lose my identity after I have the baby	4.3	13.0	**82.6**	2	0.90
Q33 After a woman has a baby, she is mainly just somebody's mother	6.2	18.6	**75.2**	2	0.83
Q34 *I am sure that I will be a good mother.	**90.1**	7.5	2.5	2	0.09
Q35 *I felt proud when I found out I was going to have a baby	**92.5**	7.5	0	2	0.67
Q36 I felt scared when I found out I was going to become a mother.	46.6	14.9	38.5	2	0.85
**Career Orientation** (Likert scale 1 to 4, underlined scores represent preference towards career orientation)	**Strongly Agree / Agree** (1-2)	**Strongly Disagree / Disagree** (3-4)	-		
Q37 *I want a job that will help me grow	**91.3**	8.8	-	3	0.55
Q38 *Being able to express myself through a job means a great deal to me.	**86.1**	13.9	-	5	0.56
Q39 *I am determined to achieve my educational and work goals	**90.5**	9.5	-	5	0.46
Q40 *Success in my work is very important to how I feel about myself	**81.6**	18.4	-	5	0.33
Q41 *I see myself as working for pay my whole adult life	**86.6**	13.4	-	6	0.46
Q42 *The responsibilities for home and family should be equally share when both partners work	**95.5**	4.5	-	7	0.23
Q43 *I need more in life than what being a wife and mother can give me	48.7	**51.3**	-	5	0.69
Q44 Women who hope to be successful in a job must do so at the expense of home and family	25.5	**74.5**	-	6	0.71
Q45 Women should seek work that will fit in family needs in terms of work hours, leave time, etc	**72.8**	27.2	-	5	0.30
Q46 Women must make changes in their careers for family needs	**71.3**	28.7	-	6	0.33
Q47 Women should not work full time when their children are young	47.8	**52.2**	-	6	0.11
Q48 Feeling loved and needed is more important to me than having a career	**84.6**	15.4	-	7	0.80
Q49 I would be very happy staying at home and not working at a job.	**55.4**	44.6	-	6	0.39

[a] Statistical significance $p<0.05$, p-value from CHI^2 test comparing sample 1 (data collection at approx. 20 weeks) and sample 2 (data collection at approx. 12 weeks); data not shown

*Questionnaire items reverse coded for sum values used in the analysis and interpretation of scales
Bold data indicates items scores ≥50%
Table adapted from: Fealy *et al.* 2020. 'Demographic and social-cognitive factors associated with gestational weight gain in an Australian pregnancy cohort', Eating Behaviors, 39: 101430.
